# Effects of Intermittent Fasting on Regulation of Metabolic Homeostasis: A Systematic Review and Meta-Analysis in Health and Metabolic-Related Disorders

**DOI:** 10.3390/jcm12113699

**Published:** 2023-05-26

**Authors:** Ana Inês Silva, Manuel Direito, Filipa Pinto-Ribeiro, Paula Ludovico, Belém Sampaio-Marques

**Affiliations:** 1 Life and Health Sciences Research Institute (ICVS), School of Medicine, University of Minho, 4710-057 Braga, Portugal; 2 ICVS/3B’s-PT Government Associate Laboratory, 4806-909 Guimarães, Portugal

**Keywords:** intermittent fasting, metabolic homeostasis, metabolic syndrome, obesity, diabetes *mellitus* type 2, adiposity, lipid homeostasis, insulin homeostasis, blood pressure

## Abstract

Intermittent fasting (IF) is an emerging dietetic intervention that has been associated with improved metabolic parameters. Nowadays, the most common IF protocols are Alternate-Day Fasting (ADF) and Time-Restricted Fasting (TRF), but in this review and meta-analysis we have also considered Religious Fasting (RF), which is similar to TRF but against the circadian rhythm. The available studies usually include the analysis of a single specific IF protocol on different metabolic outcomes. Herein, we decided to go further and to conduct a systematic review and meta-analysis on the advantages of different IF protocols for metabolic homeostasis in individuals with different metabolic status, such as with obesity, diabetes *mellitus* type 2 (T2D) and metabolic syndrome (MetS). Systematic searches (PubMed, Scopus, Trip Database, Web of Knowledge and Embase, published before June 2022) of original articles in peer-review scientific journals focusing on IF and body composition outcomes were performed. Sixty-four reports met the eligibility criteria for the qualitative analysis and forty-seven for the quantitative analysis. Herein, we showed that ADF protocols promoted the major beneficial effects in the improvement of dysregulated metabolic conditions in comparison with TRF and RF protocols. Furthermore, obese and MetS individuals are the most benefited with the introduction of these interventions, through the improvement of adiposity, lipid homeostasis and blood pressure. For T2D individuals, IF impact was more limited, but associated with their major metabolic dysfunctions—insulin homeostasis. Importantly, through the integrated analysis of distinct metabolic-related diseases, we showed that IF seems to differently impact metabolic homeostasis depending on an individual’s basal health status and type of metabolic disease.

## 1. Introduction

Intermittent fasting (IF) defines eating patterns in which individuals switch between extended periods of fasting and normal eating, on a recurring basis. IF comprises two main dietary regimens, one that includes fasting for entire days (1 to 4 per week), with a 60–100% energy restriction on fasting days (between 300 and 600 kcal) with ad libitum energy consumption on fed days, and another comprising daily time-restricted eating (fasting in a daily window, from 12 to 20 h). Essentially, these two regimens rely in approaches with both less frequent long fasting periods and short-term but frequent fasting periods [1]. Within these two main IF protocols, it is possible to describe four different types of fasting: (1) Alternate-Day Fasting (ADF) (where no food or energy-containing drinks are consumed for whole days alternating with days with ad libitum eating); (2) modified Alternate-Day Fasting regimens (mADF) (allows consumption of 20 to 25% of energy requirements on fasting days that are interspersed with days without any kind of restriction); (3) Time-Restricted Fasting (commonly TRF) (ad libitum consumption during a certain time frame during the day intercepted with a long period without any energy intake); and (4) religious fasting (RF) (fasting regimens carried out on religious or faith-based grounds). This last group includes some representative IF protocols, such as the Seventh-Day Adventists, which include the long nightly period of fasting, having the last meal of the day in the afternoon, and Ramadan fasting, in which there is no eating or drinking from sunrise to sunset, for one month, and eating occurs during the circadian sleeping phase. Nevertheless, Ramadan protocols (14 to 18 h of fasting per day), like TRF, result in a metabolic shift towards the predominant use of fatty acids as fuel, which lowers body fat [2].

The ultimate goal of IF regimens is to promote metabolic alterations with increased ketogenesis, promoting broad-spectrum benefits for health conditions, such as obesity, diabetes *mellitus*, cardiovascular disease, cancers and neurologic disorders, reviewed in [3]. The mechanisms underlying IF benefits are not solely due to upregulation of enzymes involved in ketogenesis and fatty acid oxidation (FAO), which consequently allows for the efficient utilization of these substrates as an energy source [4], but also to an overall increase in FAO associated with other metabolic adaptations [4]. The reduction in calorie intake in IF interventions also elicit an energy deficit, which consequently leads to weight and fat mass loss over time [3,4,5,6,7,8]. However, IF’s metabolic effects might be independent of weight loss due to FAO, with evidence showing that IF can improve insulin sensitivity, reduce inflammation and promote cellular repair and autophagy, which are mechanisms that help the body to cleanse and repair damaged cells [9,10]. Nevertheless, there are still contradictory observations on beneficial effects of IF that could be connected with differences between regimens. We have reviewed evidence related to the effects of different IF protocols on the regulation of metabolic homeostasis, both on healthy individuals and subjects with known metabolic dysregulation: diabetes *mellitus* type 2 (T2D), obesity and metabolic syndrome (MetS). Both T2D and MetS are closely related to obesity or to an excess of body mass index (BMI) and are also known to be prevented and/or improved by diet and lifestyle alterations. MetS is commonly described as a cluster of concomitant conditions, including increased blood pressure, high blood sugar, excess waist fat and abnormal cholesterol or triglyceride levels, increasing the risk of heart disease, stroke and T2D [11]. This systematic review and meta-analysis go further and analyses not the IF protocols but their effects on metabolic outcomes in individuals that have different metabolic status. Therefore, the primary goal was to identify benefits of IF on diverse parameters related to the optimization of cellular energy status in individuals with different requirements for metabolic homeostasis. Secondary outcomes are related to the assessment of diversity between the effects of IF on different metabolic-related disorders. We hypothesized that IF might impact adiposity, lipid homeostasis, insulin homeostasis and blood pressure differently depending on the basal body metabolic condition. This review and meta-analysis rather than analyzing IF protocols, integrates distinct metabolic disorders, highlighting the relevance of the basal body composition for the positive effects of IF. To our knowledge, this is the first review providing such a comprehensive perspective.

## 2. Materials and Methods

This systematic review and meta-analysis were conducted in accordance with the Preferred Reporting Items for Systematic Reviews and Meta-Analysis (PRISMA) statement [12]. This review’s protocol was not registered previously to its submission.

### 2.1. Search Strategies

A comprehensive literature search was conducted between November 2021 and June 2022 in the following electronic databases: PubMed, Scopus, Trip Database, Web of Knowledge and Embase using the following combination of search keywords: for PubMed—(“fasting” [MeSH Major Topic]) AND ((“obesity” [MeSH Terms]) OR (“overweight” [MeSH Terms]) OR (“metabolic syndrome” [MeSH Terms]) OR (“diabetes mellitus” [MeSH Terms]) OR (“insulin resistance” [MeSH Terms])) and, for Scopus, Trip Database, Web of Knowledge and Embase (“Intermittent fasting”) AND (“obesity” OR “overweight” OR “metabolic syndrome” OR “diabetes” OR “insulin resistance” OR “insulin sensitivity”), from 2000 up to 2022.

### 2.2. Eligibility

Original articles in peer-review scientific journals focusing on IF and body composition outcomes were retrieved. Studies were included when fulfilling the following criteria: (i) adult participants, regardless of gender; (ii) data related to adiposity (weight, body mass index (BMI), waist circumference); lipid homeostasis (HDL-c, LDL-c, total cholesterol, triglycerides), insulin homeostasis (fasting glucose, fasting insulin, insulin resistance (HOMA-IR)) or blood pressure (Systolic Blood Pressure (SBP) and Diastolic Blood Pressure (DBP)); (iii) healthy individuals and/or individuals with metabolic related disorders, such as type 2 diabetes *mellitus* (T2D), metabolic syndrome (MetS) or obesity; and, (iv) articles presenting pre- and post-intervention measures. For the meta-analysis randomized and non-randomized controlled trials, cohort studies, case-control studies and case series were included.

To avoid significant bias for the systematic review and meta-analysis, studies were excluded when one of the following criteria was observed: (i) non-original articles (systematic reviews, protocols, reviews and book chapters); (ii) articles using animal models; (iii) articles written in languages other than English; (iv) grey literature (conference abstracts, letters to editor); (v) articles where other diets or interventions were mixed, such as IF plus Mediterranean, ketogenic, paleo or vegan diets; (vi) articles on other pathologies or conditions, eating disorders, alcohol abuse, smokers, medications (especially the ones that could affect glucose and lipid profile) and pregnant or breastfeeding women; (vii) results from groups in trials that tested both IF and other diets (such as the ketogenic diet) or that had any other intervention besides IF with or without caloric restriction; (viii) studies with individuals with metabolic related disorders, such as type 2 diabetes mellitus (T2D), metabolic syndrome (MetS) or obesity associated with other severe diseases.

### 2.3. Study Selection

After removing duplicates, three researchers (AIS, MD and BSM) independently screened the title and abstract of every citation followed by full-text screening against the inclusion/exclusion criteria. To qualify for inclusion, the authors had to be in agreement.

### 2.4. Outcomes Measures

The main outcomes evaluated were the impact of IF on weight, BMI, waist circumference, HDL-c, LDL-c, total cholesterol, triglycerides, fasting glucose, fasting insulin, HOMA-IR, SBP and DBP. Although the effects of different IF protocols were not subject of analysis in this systematic review, they were briefly summarized for deeper comprehension and future reference. Different IF regimens were considered but all had to fall into one of two main groups: (1) IF with a daily window where no food or energy containing drinks were ingested or (2) whole days of the week with caloric intake greatly reduced or eliminated, as described on the characterization of the included studies.

### 2.5. Data Extraction, Management and Synthesis

Detailed data were extracted from each study and compiled in a Microsoft Excel spreadsheet by all authors, and included the following information: name of the article, authors, year of publication, the country in which the study took place, study design (randomized controlled trials (RCT), non-randomized controlled trials (NRCT), cohort studies (CS), case-control studies (CCS), case report series (CRS) and cross-section studies (CSS)), IF protocol, study duration, sample size, participant’s characteristics (gender, age and metabolic status) and outcomes measured. Data from the included studies were synthetized in the Table S1 and a narrative summary of the data is presented in the Section 3.

The meta-analysis was conducted using RevMan, version 5.4.1. Separate meta-analyses were performed for each outcome (weight, BMI, waist circumference, total cholesterol, HDL-c, LDL-c, triglycerides, fasting glucose, fasting insulin, HOMA-IR, SBP and DBP), for each IF protocol and per study group (healthy and/or individuals with type 2 diabetes *mellitus* (T2D), metabolic syndrome (MetS) or obesity).

Taking the heterogeneity of studies into consideration, a random-effects model in which the summary effect size is the mean difference of a distribution was used to aggregate data and to promote the generality of the results. Mean difference was calculated based on sample size, the mean differences (between baseline and fasting conditions) and effect direction. The 95% confidence interval (CI) and corresponding *p* values were considered as indicators of statistical significance. In an attempt to evaluate the amount of variation in the effects of included studies, we tested for heterogeneity through the (i) the Cochran’s Q statistic [13], for which a significant *p*-value (<0.05) demonstrates that studies do not share common mean differences (i.e., there is heterogeneity in the effect sizes between studies); and (ii) the I2 statistics that assess the proportion of observed dispersion that is due to real differences in the actual mean differences and is not affected by low statistical power. I2 ranges from 0 to 100%, where it is established that a value of 0% indicates no observed heterogeneity and values of 25, 50 and 75%, respectively, reflect low, moderate and high heterogeneity [13]. Group analyses were conducted to examine whether the effect of IF on body composition outcomes varied according to disease condition.

### 2.6. Quality Assessment

The risk of bias was not assessed, as this review included different type of studies. The quality of the articles was carefully assessed, according to: (i) the Critical Appraisal Skills Programme (CASP) [14] checklist for RCT, CS and CCS; (ii) the Joanna Briggs [15] checklist for CRS and CSS; and (iii) the Methodological Index for Non-Randomized Studies (MINORS) [16] checklist for NRCT. Taking into consideration that it would be nearly impossible for patients and staff of the study to be blind, items of the checklist concerning this parameter were not considered for the overall assessment of quality.

## 3. Results

### 3.1. Search Results

**Figure 1:**
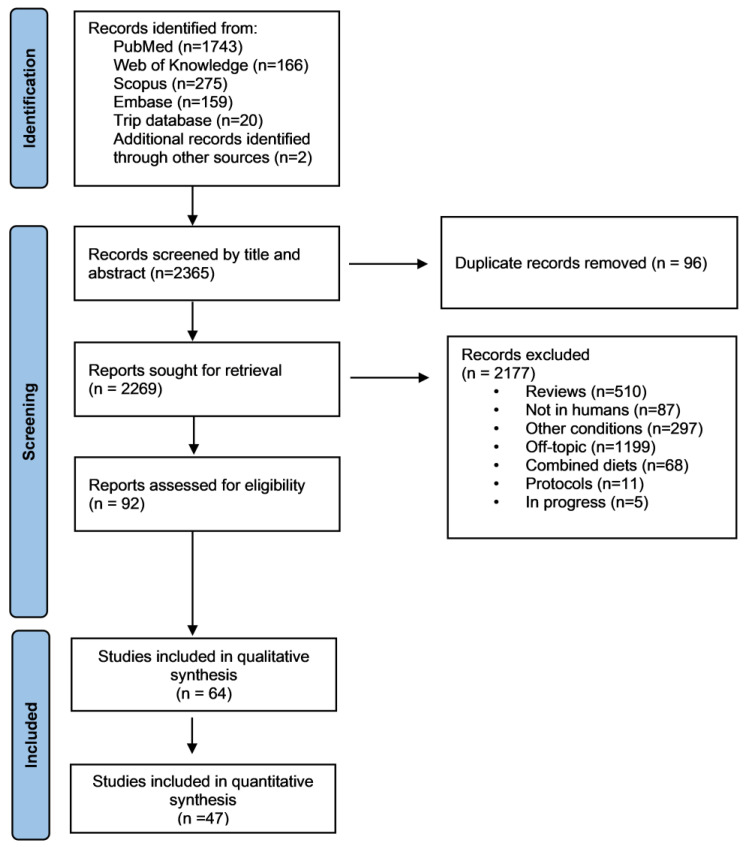
Flow chart of the methodology followed during the literature search.

PubMed, Scopus, Embase, Trip database and Web of Science generated 2365 publications (Figure 1). After the removal of 96 duplicates, 2269 publications were identified as potentially eligible. After screening the title and abstract and checking the full text for detailed information and data extraction, 64 publications were included in the systematic review (Table S1) with 47 [7,9,17,18,19,20,21,22,23,24,25,26,27,28,29,30,31,32,33,34,35,36,37,38,39,40,41,42,43,44,45,46,47,48,49,50,51,52,53,54,55,56,57,58,59,60,61] being included in the meta-analysis (Figure 1 and Table 1).

### 3.2. Characteristics of Included Studies

In the systematic review, the population comprised a total of n = 4052 participants of which n = 2007 were men and n = 1977 women. For 68 participants gender was not disclosed. Of the 64 studies, 19 included participants with T2D [6,9,17,18,20,21,37,40,42,43,46,49,57,62,63,64,65,66,67], 35 obese or overweight individuals [5,8,17,19,22,23,24,25,26,27,28,29,30,33,34,39,40,41,44,45,50,51,52,54,55,58,59,60,61,62,68,69,70,71,72,73], 7 participants with MetS [7,32,38,47,53,54,56] and 6 included healthy participants without any associated pathology [31,35,36,48,74,75] (Table S1).

Individual characteristics of the 47 studies included in the meta-analyses are summarized in Table 1, which comprises 18 randomized controlled trials (RCT), 1 non-randomized controlled trial (NRCT), 4 cohort studies (CS), 2 case control studies (CCS), 1 cross-sectional study (CSS) and 21 case-report series (CRS). The studies were conducted in distinct countries. Considering the IF protocols, we categorized them in Alternate-Day Fasting (ADF), Time-Restricted Fasting (TRF) and Religious Fasting (RF) as previously described [2]. The first group-ADF-included the following feeding + fasting protocols: 1 d + 1 d [22,29,33,55,61], 5 d + 2 d [9,25,32,54], 4 d + 3 d [23,34,47] and 1-week fasting [43]. TRF involved time restricted fasting + feeding protocols: 12 h + 12 h [26], 14 h + 10 h [56,58], 15 h + 9 h [46], 16 h + 8 h [7,19,30,35,38,41,51], 18 h + 6 h [20,24], while the RF included exclusively Ramadan fasting [17,18,21,27,28,31,36,37,39,40,42,44,45,48,49,50,52,53,57,59,60]. Importantly, ADF protocols also include the modified Alternate-Day Fasting regimens (mADF), since they may or may not include the possibility of consumption of up to 25% of the caloric needs on fasting days (between 300–600 Kcal).

### 3.3. Quality Assessment

Table S2 shows the detailed classification of each study quality according to the CASP checklist for RCT, CS and CCS; the Joanna Briggs checklist for CRS and CSS; and the MINORS checklist for NRCT. Briefly, a score of 0 points was attributed when a parameter was not met (red color), 2 points when all the criteria were fulfilled (green color) and 1 for intermediate fulfillment of criteria (yellow color). Articles evaluated with CASP checklists scoring between 0 and 10 points were considered of low quality (“weak”), from 11 to 15 of “moderate” quality, and from 16 to 20 of “good” quality. The same method as before was used the Joanna Briggs checklist for CRS. For CSS, quality was considered weak from 0 to 8, moderate from 9 to 12 and good from 13 to 16. For MINORS checklist, after eliminating the parameter assessing the blindness of the experiment, a total of 11 parameters remained. Parameters were as usual classified with a punctuation between 0 and 2 points, for a total score of 22 points. Studies ranging from 0 to 11 points were classified as low quality, between 12 and 16 of moderate quality and of good quality when a total of 17 points or more were attributed.

**Table 1:** Characterization of the studies included in the quantitative analysis synthesis.

	Study Type	Control Conditions	Experimental Conditions	IF Protocol	Duration (Days)	Age (Mean ± SD)	Participants (n and Gender)	Results Overview
1. Guo I, et al., 2021 [32]	RCT	MetS	MetS	ADF 5 + 2 (NC)	56	CTL = 42.7 ± 4.1 EG = 36.6 ± 5.7	CTL—11 M/7 W EG—10 M/12 W	Weight, waist circumference, BMI, triglycerides, insulin resistance and fasting insulin were significantly improved after IF. CTL comprises individuals with MetS maintained their habitual diet and lifestyle.
2. Parvaresh A, et al., 2019 [47]	RCT	-	MetS	ADF 4 + 3 (NC)	56	EG = 44.6 ± 9.8	EG—21 M/14 W	mADF diet is an effective option for managing body weight, waist circumference, systolic blood pressure and fasting plasma glucose when compared with common calorie restriction. CTL comprises individuals with MetS that consumed 75% of their energy needs each day.
3. Gabel K, et al., 2019 [29]	RCT	-	Obesity	ADF 1 + 1 (NC)	365	CTL = 43 ± 1 EG = 43 ± 3	CTL—4 M/11 W EG—9 M/2 W	ADF may produce greater reductions in fasting insulin and insulin resistance compared with caloric restriction in insulin-resistant participants despite similar decreases in body weight. CTL comprises obese individuals that maintained their usual eating and activity habits.
4. Cho A-Ra, et al., 2019 [23]	RCT	-	Overweight	ADF 4 + 3 (NC)	56	CTL = 42.6 ± 10.6 EG = 33.5 ± 5	CTL—3 M/2 W EG—3 M/6 W	Exercise, with or without ADF, improves cholesterol metabolism (serum sterol signatures) and increased physical activity has a greater effect on cholesterol biosynthesis than weight reduction or calorie restriction. CTL comprises overweight individuals that maintained their usual eating and activity habits.
5. Corley BT, et al., 2018 [9]	RCT	-	T2D	ADF 5 + 2 (C) 5 + 2 (NC)	84	62 (44 to 77) * 58 (42 to 74) *	11 M/7 W 11 M/8 W	IF in a 5 + 2 protocol (both consecutive and non-consecutive) increases the rate of hypoglycaemia in patients with T2D, even after education on this topic and medication adjustments. Yet it improves weight, HbA1c, fasting glucose and quality of life (both protocols).
6. Hutchison AT, et al., 2018 [34]	RCT	Overweight or Obesity	Overweight or Obesity	ADF 4 + 3 (NC)	70	CTL 1 = 49 ± 3 CTL 2 = 49 ± 2 EG-IF100 = 51 ± 2 EG-IF70 = 51 ± 2	CTL 1—11 W CTL 2—11 W EG-IF100—22 W EG-IF70—22 W	IF combined with a 30% caloric restriction is better at improving weight, fat mass, HOMA-IR, fasting glucose, non-esterified fatty acids, total cholesterol, LDL-cholesterol and triglycerides than energy-matched continuous caloric restriction. CTL comprises overweight or obese women maintained under continuous food intake at 100% of baseline energy requirements.
7. Sundfør TM, et al., 2018 [54]	RCT	-	Overweight and MetS	ADF 5 + 2 (NC)	365	EG = 49.9 ± 10.1	EG—28 M/26 W	Both intermittent and continuous energy restriction promoted similar weight loss, maintenance and improvement in cardiovascular risk factors after one year. However, the feelings of hunger, appears to be more pronounced during IF.
8. Trepanowski JF, et al., 2018 [61]	RCT	-	Obesity	ADF 1 + 1 (NC)	168	CTL = 44 ± 2 EG = 46 ± 2	CTL—4 M/21 W EG—3 M/22 W	ADF and CR similarly increased the fat-free mass:total-mass ratio, decreased circulating leptin and did not affect the visceral adipose tissue:subcutaneous adipose tissue ratio or other measured adipokines. Weight loss, rather than the pattern of energy restriction, appeared to be the main driver of these changes. CTL comprises obese individuals that maintained their usual diet.
9. Conley M, et al., 2018 [25]	RCT	-	Obesity	ADF 5 + 2 (NC)	180	EG = 68 ± 2.7	EG—11 M	The 5:2 diet is a feasible weight loss strategy in this older male population. Furthermore, it also indicates that participants were able to follow the diet sufficiently to induce magnitudes of weight loss similar to that of standard dietary modification practices, and the diet did not appear to cause an unbalanced nutritional intake.
10. Li C, et al., 2017 [43]	RCT	T2D	T2D	ADF 1 week fasting	7	CTL = 64.4 ± 5.7 EG = 64.7 ± 7	CTL—16 NS EG—16 NS	A 1-week fasting therapy is promising for the improvement of weight, waist circumference, SBP, DBP and HOMA-IR in T2D patients, when compared with typical T2D medication. CTL comprises T2D individuals that followed the principles of a Mediterranean diet.
11. Catenacci VA et al., 2016 [22]	RCT	-	Obesity	ADF 1 + 1 (NC)	56	EG = 39.6 ± 9.5	EG—3 M/10 W	ADF is a safe and tolerable approach for weight loss, it improved weight, body composition, lipids and insulin sensitivity index at 8 weeks while not increasing the risk for weight regain up-to 24 weeks after completing the intervention.
12. Hoddy KK, et al., 2014 [33]	RCT	-	Obesity	ADF 1 + 1 (NC)	70	ADF-L: 45 ± 3 ADF-D: 45 ± 3 ADF-SM: 46 ± 2	ADF-L: 3 M/17 W ADF-D: 4 M/15 W ADF-SM: 2 M/18 W	This study demonstrated there is flexibility in the timing of the fast day meal during ADF. People with obesity may feed at dinner or as small meals throughout the day, and experience similar weight loss, body composition and cardiovascular benefits as the traditional lunch time approach.
13.Varady KA et al., 2009 [55]	CRS	-	Obesity	ADF 1 + 1 (NC)	56	EG = 46 ± 2.4	EG—4 M/12 W	ADF is a viable diet option to help obese individuals lose weight and decrease coronary artery disease risk.
1. Kotarsky CJ, et al., 2021 [41]	RCT	Overweight or Obesity	Overweight or Obesity	TRF 16 + 8	56	CTL = 44 ± 2 EG = 45 ± 3	CTL—9 M/1 W EG—9 M/2 W	IF associated with exercise training is a short-term dietary strategy for reducing fat mass and increasing lean mass in overweight and obese adults. CTL comprises overweight or obese individuals that maintained their usual diet.
2. de Oliveira Maranhao Pureza IR, et al., 2021 [26]	RCT	Overweight or Obesity	Overweight or Obesity	TRF 12 + 12	365	19–44	EG –31 W	IF may help in the long-term management of obesity.
3. Kunduraci YE, et al., 2020 [7]	RCT	MetS	MetS	TRF 16 + 8	84	EG = 47.44 ± 2.17	EG—16 M/16 W	Weight, BMI, total cholesterol, LDL, triglycerides, fasting glucose, systolic and diastolic blood pressure, insulin resistance and fasting insulin were significantly improved after IF.
4. Cienfuegos S, et al., 2020 [24]	RCT	-	Obesity	TRF 20 + 4 18 + 6	56	4 h_TRF: 49 ± 2 6 h_TRF: 46 ± 3	4 h_TRF: 2 M/14 W 6 h_TRF: 1 M/18 W	4- and 6-h TRF regimens lead to similar weight loss over the 2 months in peolple with obesity while also decreasing insulin resistance and oxidative stress. CTL comprises obese individuals that maintained their usual diet.
5. Jones R, et al., 2020 [35]	CCS	Healthy	Healthy	TRF 16 + 8	14	CTL = 23 ± 1 EG = 23 ± 1	CTL—8 M EG—8 M	Weight showed significant improvement after IF. CTL comprises healthy individuals following a dietary plan provided with all food and beverages that matched the macronutrient composition (45% CHO, 35% fat and 20% protein).
6. Zhao L, et al., 2022 [58]	CRS		Overweight	TRF 14 + 10	56	63 ± 4	15 M	This study demostrated that TRF had a net effect of reducing glycemia and dampening energy-consuming pathways in adipose tissue.
7. Parr EB, et al., 2020 [46]	CRS	T2D	T2D	TRF 15 + 9	28	50.2 ± 8.9	9 M/10 W	Fasting insulin showed significant improvements after IF.
8. Gabel K, et al., 2020 [30]	CRS	-	Obesity	TRF 16 + 8	84	-	14 M	This study suggest that the mild weight loss (2%) induced by time restricted eating did not significantly alter the diversity or overall composition of the gut microbiome.
9. Wilkinson MJ, et al., 2020 [56]	CRS	-	MetS	TRF 14 + 10	84	59 ± 11.4	15 M/6 W	TRF mproved cardiometabolic health of patients with metabolic syndrome receiving standard medical care including high rates of statin and anti-hypertensive use.
10. Anton SD, et al., 2019 [19]	CRS	-	Overweight	TRF 16 + 8	21	77.1	4 M/6 W	TRF is an acceptable and feasible eating pattern for overweight, older adults to follow while also promoting short-term weight loss.
11. Kesztyüs D, et al., 2019 [38]	CRS	-	MetS	TRF 16 + 8	90	49.1± 12,4	9 M/31 W	TRE may help to reduce abdominal obesity and hence prevent cardio-metabolic diseases.
12. Arnason FB, et al., 2017 [20]	CRS	-	T2D	TRF 18–20 h fasting per day	14	53.8 ± 9.11	1 M/9 W	Weight and BMI were improved after IF.
13. Schroder JD, et al., 2021 [51]	NRCT	Obesity	Obesity	TRF 16 + 8	90	CTL = 42.3 ± 3.5 EG = 36.6 ± 1.6	CTL—12 W EG—20 W	Weight, waist circumference, BMI, SBP and DBP were improved after IF. CTL comprises obese individuals that maintained their usual diet.
1. Zouhal H, et al., 2020 [59]	RCT	-	Obesity	RF Ramadan	30	CTL = 23.8 ± 3.8 EG = 24 ± 3.4	CTL—14 M EG—14 M	Ramadan fasting improves systemic inflammation biomarkers in males with obesity. CTL comprises obese individuals that did not fast during Ramadan.
2. Zouhal H, et al., 2020 [60]	RCT	-	Obesity	RF Ramadan	30	CTL = 23.8 ± 3.7 EG = 24.5 ± 3.8	CTL—15 M EG—15 M	IF during Ramadan is an effective strategy to modify appetite-regulating hormones, leading to improved body composition indices and reduced obesity.
3. Abdullah K, et al., 2020 [18]	CS	Healthy	Healthy or T2D	RF Ramadan	30	CTL = 34.61 ± 4.31 EG = 34.35 ± 3.83 EG = 50.17 ± 12.95	CTL—31 M EG—37 M EG—30 M	This study compares the effect of IF in patients with T2D, their first-degree relatives and healthy individuals. Leptin, adiponectin, leptin:adiponectin ratio, HOMA-beta and HbA1c were significantly improved in all groups. Fasting blood glucose and growth hormone levels were improved in control and first-degree relatives. C-peptide, HOMA-IR, HOMA-S and insulin levels were improved in T2D patients and first-degree relatives. CTL comprises healthy control with fasting blood glucose <100 mg/dL.
4. Yeoh ECK, et al., 2015 [57]	CS		T2D	RF Ramadan	30	57 ± 11	15 M/14 W	Ramadan fasting can be practiced safely with prior patient education and medication adjustment with modest benefits on metabolic profile and body composition, particularly among females.
5. Feizollahzadeh S, et al., 2014 [28]	CS	-	Healthy with Overweight	RF Ramadan	30	47.88	70 M	Weight and BMI were improved after IF although total cholesterol, triglycerides and fasting glucose levels were also increased.
6. Karatoprak C, et al., 2013 [37]	CS		T2D	RF Ramadan	30	57.4 ± 10.1	19 M/57 W	Results showed no negative effects of extended fasting on glucose regulation in diabetic patients using certain medications.
7. McNeil J, et al., 2014 [45]	CCS	Healthy	Obesity	RF Ramadan	14	27 ± 4.5	10 M	Data demostrated significant increased of glucose, total cholesterol and LDL-C levels during Ramadan fast in normal-weight and obese men. Dietary restraint scores were also greater during Ramadan. Lastly, changes in anthropometric parameters were related to changes in metabolic profiles, dietary restraint and disinhibition eating behavior trait scores.
8. Kovil R, et al., 2020 [42]	CRS	-	T2D	RF Ramadan	30	21–80 **	25 M/25 W	This study did not show any significant changes in the parameters evaluated after IF.
9. Faris E, et al., 2019 [27]	CRS	-	Overweight or Obesity	RF Ramadan	30	36.2 ± 12.5	35 M/22 W	Weight, BMI, total cholesterol, triglycerides and SBP were improved after IF although HDL was also decreased.
10. Madkour MI, et al., 2019 [44]	CRS	-	Healthy or Obesity	RF Ramadan	30	CTL = 29.8 ± 14 EG = 35.72 ± 12.35	CTL—6 EG—34 M/22 W	Fasting glucose, insulin, insulin resistance expressed in homeostatic model assessment (HOMA-IR) remained unchanged throughout the study, while significant (*p* < 0.05) decreases in total cholesterol, triglycerides and HDL cholesterol were observed.
11. Abdessadek M, et al., 2019 [17]	CRS	-	T2D	RF Ramadan	30	-	57 M/93 W	This study showed a significant decrease in glycemic parameters (glycated haemoglobin and fasting blood glucose), and also significant variations in lipid profile before and after Ramadan, respectively. Furthermore, it also demostrated that in well-controlled T2D patients under antidiabetic drugs, the risk of hypoglycaemia is very low and patients may fast safely in Ramadan.
12. Prasetya G, et al., 2018 [48]	CRS	-	Healthy	RF Ramadan	29	24.3 ± 3.7	27 M	Weight, waist circumference, BMI, insulin resistance and fasting insulin were improved after IF although HDL levels were also decreased.
13. Kamble S, et al., 2018 [36]	CRS	-	Healthy	RF Ramadan	39	20–35 **	30 (NS)	No changes after IF.
14. Sezen Y, et al., 2016 [52]	CRS	-	Obesity	RF Ramadan	30	37 ± 7	70 M	Ramadan fasting was beneficial for body composition, but had no effect on arterial stiffness and resting heart rate.
15. Gnanou JV, et al., 2015 [31]	CRS	-	Healthy	RF Ramadan	39	19–23 **	20 M	Weight, BMI, fasting glucose, fasting insulin and insulin resistance were improved after IF.
16. Sahin SB, et al., 2013 [49]	CRS	-	T2D	RF Ramadan	30	56.93 ± 9.57	40 M/82 W	Fasting during Ramadan did not worsen the glycemic control of T2D patients.
17. Shariatpanahi MV, et al., 2012 [53]	CRS	-	MetS	RF Ramadan	30	40.14 ± 10.8	65 M	Change in the number and timing of meals and portioning of the daily energy consumption decreases inflammatory markers in MetS.
18. Salehi M and Neghab M, 2007 [50]	CRS	-	Obesity	RF Ramadan	29	23.4 ± 1.3	28 M	Consumption of a medium calorie balanced diet in conjunction with sufficient fluid intake during Ramadan and fasting may significantly decrease serum levels of glucose, cholesterol, as well as weight and BMI.
19. Khaled MB, et al., 2006 [39]	CRS	-	Obesity	RF Ramadan	29	23.4 ± 1.3	60 W	Beneficial effect of Ramadan fasting on glucose homeostasis, and an unbalanced profile on lipids.
20. Khatib FA and Shafagoj YA, 2004 [40]	CRS	-	T2D/Obesity	RF Ramadan	29	51 ± 10	44 M	Non-insulin dependent T2D patients displayed a trend towards better glycemic control following Ramadan fasting.
21. Bener A, et al., 2018 [21]	CSS		T2D	RF Ramadan	30	55.39 ± 15.3	593 M/653 W	Ramadan fasting has positive effects on T2D patients by decreasing blood pressure, blood glucose and HbA1C levels and BMI. It also improved sleep duration and physical activity.
**TOTAL**							**1551 M/1491 W/** **68 NS**	

RCT—Randomized controlled trial; CS—Cohort study; CRS—Case report series; NRCT—non-randomized controlled trial; CSS—Cross-sectional study; MetS—Metabolic syndrome; T2D—diabetes mellitus type 2; C—consecutive; NC—Non-consecutive; CTL—Control; EG—Experimental Group; NS—Not specified; ADF—alternate-day eating; CR—caloric restriction; TRF—Time-restrict eating; IF70—Intermittent fasting 70%; IF100—Intermittent fasting 100%; M—Men; W—Women; HOMA-IR—insulin resistance; BMI—Body mass index; SBP—Systolic blood pressure; DBP—Diastolic blood pressure; * mean (range); ** range.

Of the 64 studies identified as relevant for this systematic review, the overall methodological quality of 45 was rated as “good” (70.3%), 17 studies as “moderate” (26.5%) and 2 as “weak” (3.1%). Overall, the quality of studies was good, and Table S2 summarizes the proportion of studies that met each criterion. Independently of the study type, the studies that presented good quality met criteria including, per example, the clarity of the study objective, the study randomization, the implications of the studies for practice, among others. The criteria that most of the studies failed to meet was the identification of confounding factors, as well as its interference in the study design, the groups’ randomization and the lack of demographic data (Table S2).

### 3.4. Meta-Analysis

The meta-analysis results of the effects of IF on the regulation of metabolic homeostasis in health and disease (T2D, MetS and obesity) were based on data from 47 studies selected considering the completeness of the outcomes measured in four main categories: adiposity (weight, BMI, waist circumference), lipid homeostasis (HDL-c, LDL-c, total cholesterol, triglycerides), insulin homeostasis (fasting glucose, fasting insulin, HOMA-IR) and blood pressure (SBP and DBP). According to IF protocols, these metabolic parameters were evaluated independently in 13 ADF, 13 TRF and 21 RF studies.

### 3.5. Characterization of Participants Included in Each IF Protocol

Changes in body weight, BMI, waist circumference, glucose and total cholesterol of the participants included in this meta-analysis are reported in Table 2. Overall, weight loss, BMI, waist circumference and total cholesterol levels were similar among participants included in each of the IF regimes. Nevertheless, we noticed that participants from RF studies presented higher glucose levels in comparison with the participants that integrate the ADF and TRF studies. This difference could be related to the fact that most of the RF studies were performed with T2D individuals, that by itself present higher glucose levels.

### 3.6. Effects of IF on Regulation of Metabolic Homeostasis

#### 3.6.1. Adiposity

**Figure 2:**
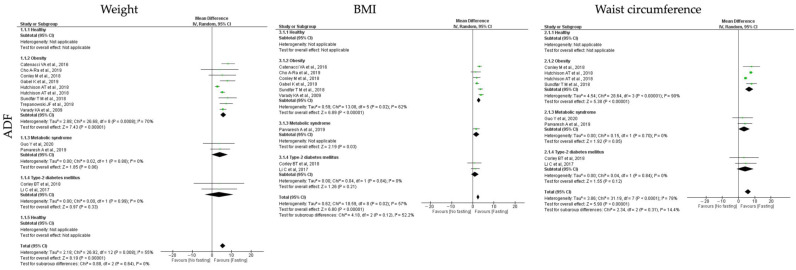
Forest plot of the data from random effects meta-analysis shown as mean difference with 95% confidence intervals on adiposity outcomes, weight, body mass index (BMI) and waist circumference, for the studies that presented data concerning these parameters. Data presented are related with alternate-day fasting (ADF) and the metabolic conditions: healthy or disease condition, obesity, T2D or MetS. For each study, the square represents the mean difference between baseline and fasting conditions, with the horizontal line intersecting it as the lower and upper limits of the 95% confidence interval [9,22,23,25,29,32,34,43,47,54,55,61].

**Figure 3:**
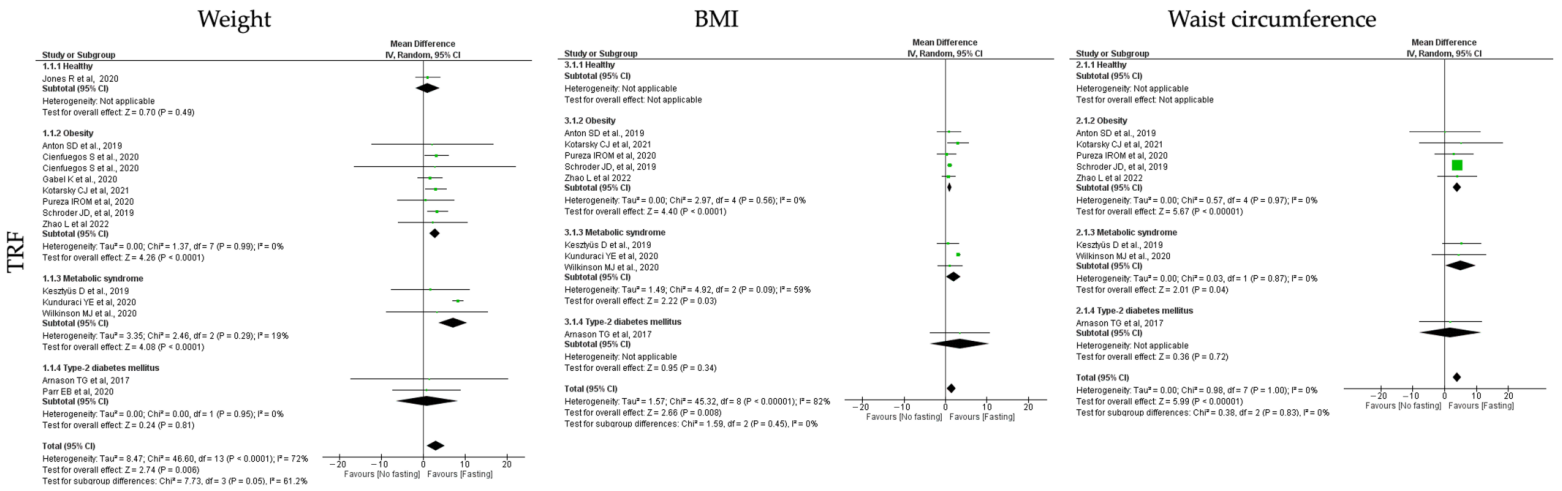
Forest plot of the data from random effects meta-analysis shown as mean difference with 95% confidence intervals on adiposity outcomes, weight, body mass index (BMI) and waist circumference, for the studies that presented data concerning these parameters. Data presented are related with time-restricted fasting (TRF) and the metabolic conditions: healthy or disease condition, obesity, T2D or MetS. For each study, the square represents the mean difference between baseline and fasting conditions, with the horizontal line intersecting it as the lower and upper limits of the 95% confidence interval [7,19,20,24,26,30,35,38,41,46,51,56,58].

**Figure 4:**
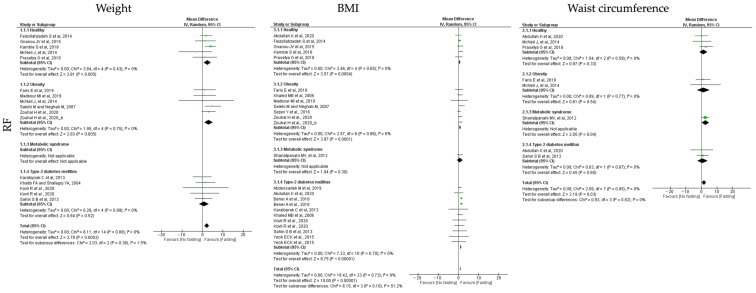
Forest plot of the data from random effects meta-analysis shown as mean difference with 95% confidence intervals on adiposity outcomes, weight, body mass index (BMI) and waist circumference, for the studies that presented data concerning these parameters. Data presented are related with and religious fasting (RF) and the metabolic conditions: healthy or disease condition, obesity, T2D or MetS. For each study, the square represents the mean difference between baseline and fasting conditions, with the horizontal line intersecting it as the lower and upper limits of the 95% confidence interval [17,18,21,27,28,31,36,37,39,40,42,44,45,48,49,50,52,53,57,59,60].

Concerning the adiposity measurements, the analyzed outcomes were weight, BMI and waist circumference. In general, all the tested IF protocols (ADF, TRF and RF) showed significant positive results for the analyzed outcomes, when comparing pre- and post-fasting timepoints (Table 3, Table 4 and Table 5; Figure 2, Figure 3 and Figure 4).

For **ADF protocols**, a total of 13 studies were included and data showed a significant reduction in the body weight (k = 12, 5.54 (95% CI [4.21, 6.86]), *p* < 0.00001), BMI (k = 9, 2.50 (95% CI [1.78, 3.21]), *p* < 0.00001) and waist circumference (k = 7, 5.80 (95% CI [3.87, 7.72]), *p* < 0.00001), with high heterogeneity between studies (Q = 26.92, *p* = 0.008, I2 = 55.00%; Q = 18.59, *p* = 0.05, I2 = 57.00% and Q = 31.19, *p* < 0.00001, I2 = 78.00%, respectively) (Table 3; Figure 2).

A deeper analysis throughout stratification of the different metabolic conditions, people with obesity, T2D and MetS individuals, revealed that in relation to weight, ADF interventions presented statistical positive effects only for people with obesity (k = 8, 5.82 (95% CI [4.29, 7.36]), *p* < 0.00001), with high heterogeneity between studies (Q = 26.68, *p* = 0.0008, I2 = 70.00%) (Table 3; Figure 2).

Concerning BMI, analysis showed that obese and MetS individuals benefit with the ADF intervention (k = 6, 2.83 (95% CI [2.03, 3.64]), *p* < 0.00001; k = 1, 1.70 (95% CI [0.18, 3.22]), *p* = 0.03, respectively). Heterogeneity was high for the people with obesity (Q = 13.08, *p* = 0.02, I2 = 62.00%) (Table 3; Figure 2).

From the seven studies used to estimate the impact of ADF protocols in waist circumference, data showed that ADF decreases abdominal circumference of obese and MetS individuals (k = 3, 6.67 (95% CI [4.24, 9.10]), *p* < 0.00001; k = 2, 3.52 (95% CI [−0.06, 7.10]), *p* = 0.05). Studies with obese people displayed high heterogeneity (Q = 28.64, *p* < 0.00001, I2 = 90.00%), while low heterogeneity was observed for studies performed with MetS individuals (Q = 0.15, *p* = 0.70, I2 = 0.00%) (Table 3; Figure 2). It is worth noting that with the ADF studies no data related with healthy group were found.

Regarding the analysis of **TRF** protocol, the 13 assessed studies demonstrated a significant reduction in the body weight (k = 13, 3.05 (95% CI [0.87, 5.23]), *p* = 0.006), BMI (k = 9, 1.48 (95% CI [0.39, 2.57]), *p* = 0.008) and waist circumference (k = 8, 3.93 (95% CI [2.64, 5.21]), *p* < 0.00001), with high heterogeneity between studies for weight and BMI (Q = 46.60, *p* < 0.00001, I2 = 72.00% and Q = 45.32, *p* < 0.00001, I2 = 82.00%, respectively) and low heterogeneity for waist circumference studies (Q = 0.98, *p* = 1.00, I2 = 0.00%) (Table 4; Figure 3).

Specific analysis of each outcome showed that TRF significantly decreases the weight of obese (k = 7, 2.78 (95% CI [1.50, 4.06]), *p* < 0.00001) and MetS individuals (k = 3, 7.14 (95% CI [3.71, 10.57]), *p* < 0.00001). Heterogeneity was low in the obesity and MetS groups (Q = 1.39, *p* = 0.99, I2 = 0.00% and Q = 2.46, *p* = 0.29, I2 = 19.00%, respectively) (Table 4; Figure 3).

Studies demonstrated that TRF protocols significantly improved BMI in obese (k = 5, 1.01 (95% CI [0.56, 1.46]), *p* < 0.00001) and MetS (k = 3, 2.01 (95% CI [0.24, 3.78]), *p* = 0.03) individuals. Heterogeneity was low in obesity group (Q = 2.97, *p* = 0.56, I2 = 0.00%) and moderate for the MetS group (Q = 4.92, *p* = 0.09, I2 = 59.00%) (Table 4; Figure 3).

TRF protocols promoted a statistically significant reduction in waist circumference in individuals with obesity (k = 5, 3.89 (95% CI [2.54, 5.23]), *p* < 0.00001) and MetS (k = 2, 5.01 (95% CI [0.12, 9.90]), *p* = 0.04). Low heterogeneity was observed between the studies analyzed for the people with obesity (Q = 0.57, *p* = 0.97, I2 = 0.00%) or MetS (Q = 0.03, *p* = 0.87, I2 = 0.00%) (Table 4; Figure 3).

**Table 3:** Analysis of the impact of alternate-day fasting (ADF) protocols on different outcomes in healthy individuals and/or individuals with metabolic related disorders as type 2 diabetes *mellitus* (T2D), metabolic syndrome (MetS) or obesity.

	Moderators	k ^1^	Point Estimate	CI Lower	CI Upper	*p*-Value	Heterogeneity		
							Q-Value	*p*-Value	I-Squared
**Adiposity**	Weight (kg)								
	All studies	12	5.54	4.21	6.86	<0.00001	26.92	0.008	55.00
	Healthy	0	-	-	-	-	-	-	-
	Obesity	8	5.82	4.29	7.36	<0.00001	26.68	0.0008	70.00
	T2D	2	3.67	−3.71	11.04	0.33	0.00	0.99	0.00
	MetS	2	4.00	−0.23	8.24	0.06	0.02	0.88	0.00
	BMI (kg/m^2^)								
	All studies	9	2.50	1.78	3.21	<0.00001	18.59	<0.02	57.00
	Healthy	0	-	-	-	-	-	-	-
	Obesity	6	2.83	2.03	3.64	<0.00001	13.08	0.02	62.00
	T2D	2	1.09	−0.61	2.79	0.21	0.04	0.84	0.00
	MetS	1	1.70	0.18	3.22	0.03	-	-	-
	Waist circumference (cm)								
	All studies	7	5.80	3.87	7.72	<0.00001	31.19	<0.00001	78.00
	Healthy	0	-	-	-	-	-	-	-
	Obesity	3	6.67	4.24	9.10	<0.00001	28.64	<0.00001	90.00
	T2D	2	4.12	−1.09	9.33	0.12	0.04	0.84	0.00
	MetS	2	3.52	−0.06	7.10	0.05	0.15	0.70	0.00
**Lipid homeostasis**	HDL-c (mg/dL)								
	All studies	11	1.49	0.72	2.26	0.0002	803.57	<0.00001	98.00
	Healthy	0	-	-	-	-	-	-	-
	Obesity	8	1.59	0.79	2.39	<0.00001	796.57	<0.00001	99.00
	T2D	2	−0.51	−4.25	3.24	0.79	0.84	0.36	0.00
	MetS	1	1.00	−2.92	4.92	0.62	-	-	-
	LDL-c (mg/dL)								
	All studies	12	7.06	3.19	10.93	0.0004	22,243.31	<0.00001	100.00
	Healthy	0	-	-	-	-	-	-	-
	Obesity	8	7.67	3.51	11.83	0.0003	22,238.46	<0.00001	100.00
	T2D	2	3.49	−12.02	19.00	0.66	0.01	0.94	0.00
	MetS	2	3.10	−7.45	13.65	0.58	0.39	0.53	0.00
	Total cholesterol (mg/dL)								
	All studies	12	13.16	3	17.16	<0.00001	22,070.51	<0.00001	100.00
	Healthy	0	-	-	-	-	-	-	-
	Obesity	8	13.87	9.62	18.12	<0.00001	22,062.59	<0.00001	100.00
	T2D	2	8.84	−7.79	25.48	0.30	0.77	0.38	0.00
	MetS	2	7.39	−5.11	19.90	0.25	0.52	0.47	0.00
	Triglycerides (mg/dL)								
	All studies	10	26.17	−0.62	55.45	0.06	1134.75	<0.00001	99.00
	Healthy	0	-	-	-	-	-	-	-
	Obesity	7	25.38	−4.68	55.45	0.10	1128.37	<0.00001	99.00
	T2D	2	11.20	−15.84	38.24	0.42	0.23	0.63	0.00
	MetS	1	52.00	9.41	94.59	0.02	-	-	-
**Insulin homeostasis**	Fasting glucose (mg/dL)								
	All studies	10	4.33	−1.14	9.79	0.12	99,283	<0.00001	99.00
	Healthy	0	-	-	-	-	-	-	-
	Obesity	7	1.14	−1.89	4.17	0.46	157.83	<0.00001	96.00
	T2D	2	19.17	14.63	23.72	<0.00001	1.15	0.28	13.00
	MetS	1	5.00	0.71	9.29	0.02	-	-	-
	Fasting insulin (mU/L)								
	All studies	8	2.55	−0.88	5.98	0.15	330.18	<0.00001	98.00
	Healthy	0	-	-	-	-	-	-	-
	Obesity	5	2.36	−1.76	6.48	0.26	329.38	<0.00001	98.00
	T2D	1	3.70	−5.31	12.71	0.42	-	-	-
	MetS	2	2.54	−0.08	5.15	0.06	0.06	0.80	0.00
	HOMA-IR								
	All studies	6	0.90	−0.14	1.95	0.09	329.27	<0.00001	98.00
	Healthy	0	-	-	-	-	-	-	-
	Obesity	4	0.88	−0.33	2.10	0.16	328.54	<0.00001	99.00
	T2D	1	1.50	−1.63	4.63	0.53	-	-	-
	MetS	1	0.73	−0.01	1.47	<0.00001	-	-	-
**Blood pressure**	SBP (mmHg)								
	All studies	8	6.08	4.08	8.08	<0.00001	24.27	0.004	63.00
	Healthy	0	-	-	-	-	-	-	-
	Obesity	4	5.49	3.41	7.57	<0.00001	17.68	0.003	72.00
	T2D	2	8.21	−1.38	17.80	0.09	1.83	0.18	45.00
	MetS	2	10.46	2.08	18.83	0.01	1.58	0.21	37.00
	DBP (mmHg)								
	All studies	8	3.52	2.55	4.49	<0.00001	17.14	0.05	48.00
	Healthy	0	-	-	-	-	-	-	-
	Obesity	4	3.23	2.32	4.13	<0.00001	10.19	0.07	51.00
	T2D	2	5.79	−0.08	11.65	0.05	1.39	0.24	28.00
	MetS	2	5.76	0.30	11.22	0.04	2.09	0.15	52.00

^1^ k—number of studies; MetS—Metabolic syndrome; T2D—diabetes *mellitus* type 2; HOMA—insulin resistance; BMI—Body mass index; SBP—Systolic Blood Pressure; DBP—Diastolic Blood Pressure.

Regarding **RF** interventions, results revealed less impact on the adiposity outcomes when compared with ADF or TRF protocols. The RF studies revealed positive effects on reduction in weight in healthy (k = 5, 2.44 (95% CI [0.74, 4.14]), *p* = 0.005) and obese individuals (k = 6, 3.01 (95% CI [0.92, 5.10]), *p* = 0.005). Low heterogeneity was observed between the studies analyzed in each group (Table 5; Figure 4).

RF improved BMI in healthy (k = 5, 0.70 (95% CI [0.32, 1.09]), *p* = 0.0004), obese (k = 7, 0.84 (95% CI [0.43, 1.26]), *p* < 0.00001) and T2D (k = 8, 1.26 (95% CI [0.98, 1.54]), *p* < 0.00001) individuals. Heterogeneity was low in the healthy, obesity and T2D groups (Q = 2.46, *p* = 0.65, I2 = 0.00%; Q = 2.57, *p* = 0.86, I2 = 0.00% and Q = 7.23, *p* = 0.70, I2 = 0.00%, respectively (Table 5; Figure 4).

Concerning waist circumference, only MetS individuals revealed statistical reduction in waist circumference with RF (k = 1, 2.61 (95% CI [0.12, 5.10]), *p* = 0.04), with low heterogeneity between the analyzed studies (Table 5; Figure 4).

#### 3.6.2. Lipid Homeostasis

**Figure 5:**
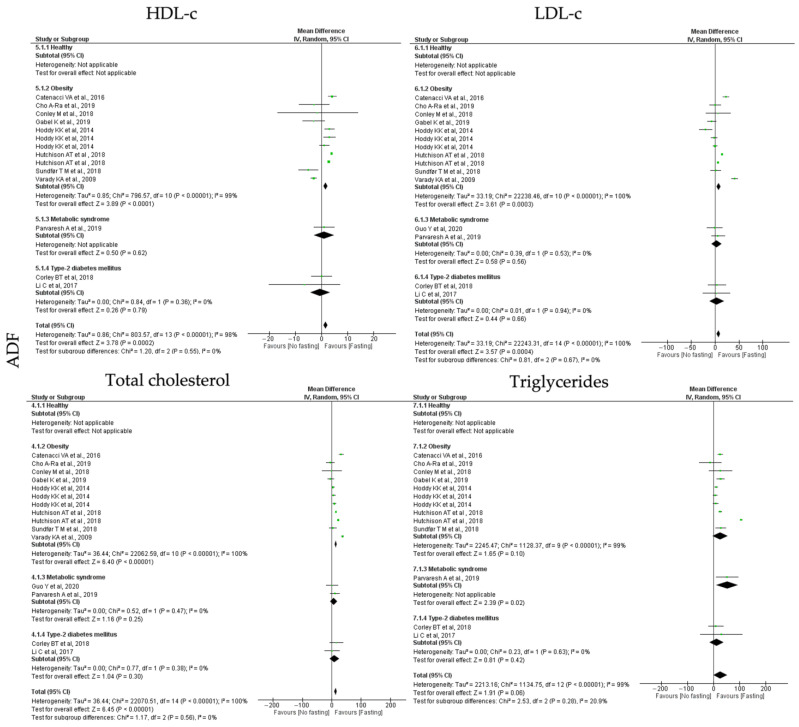
Forest plot of the data from random effects meta-analysis shown as mean difference with 95% confidence intervals on lipid homeostasis outcomes, HDL-c, LDL-c, total cholesterol, triglycerides, for the studies that presented data concerning these parameters. Data are presented according to alternate-day fasting (ADF) and the metabolic conditions: healthy or disease condition, obesity, T2D or MetS. For each study, the square represents the mean difference between baseline and fasting conditions, with the horizontal line intersecting it as the lower and upper limits of the 95% confidence interval [9,22,23,25,29,32,33,34,43,47,54,55].

**Figure 6:**
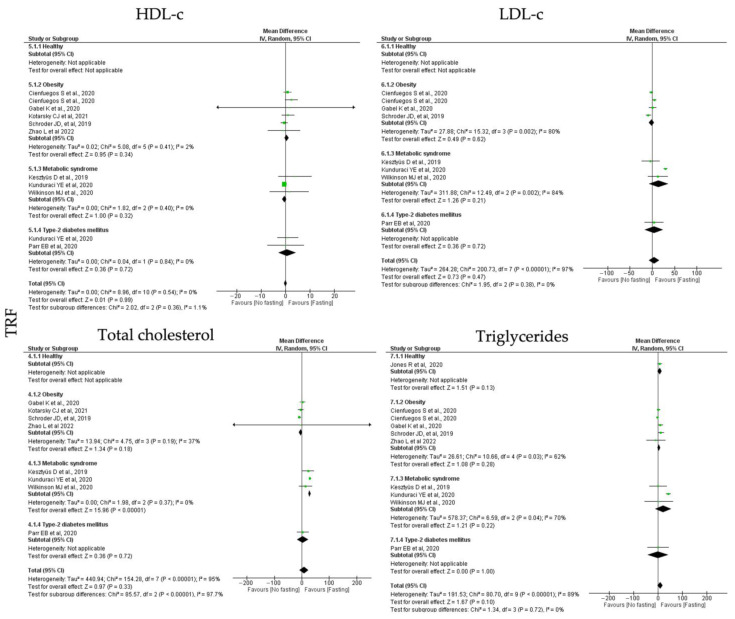
Forest plot of the data from random effects meta-analysis shown as mean difference with 95% confidence intervals on lipid homeostasis outcomes, HDL-c, LDL-c, total cholesterol, triglycerides, for the studies that presented data concerning these parameters. Data are presented according to time-restricted fasting (TRF) and the metabolic conditions: healthy or disease condition, obesity, T2D or MetS. For each study, the square represents the mean difference between baseline and fasting conditions, with the horizontal line intersecting it as the lower and upper limits of the 95% confidence interval [7,24,30,38,41,46,51,56,58].

**Figure 7:**
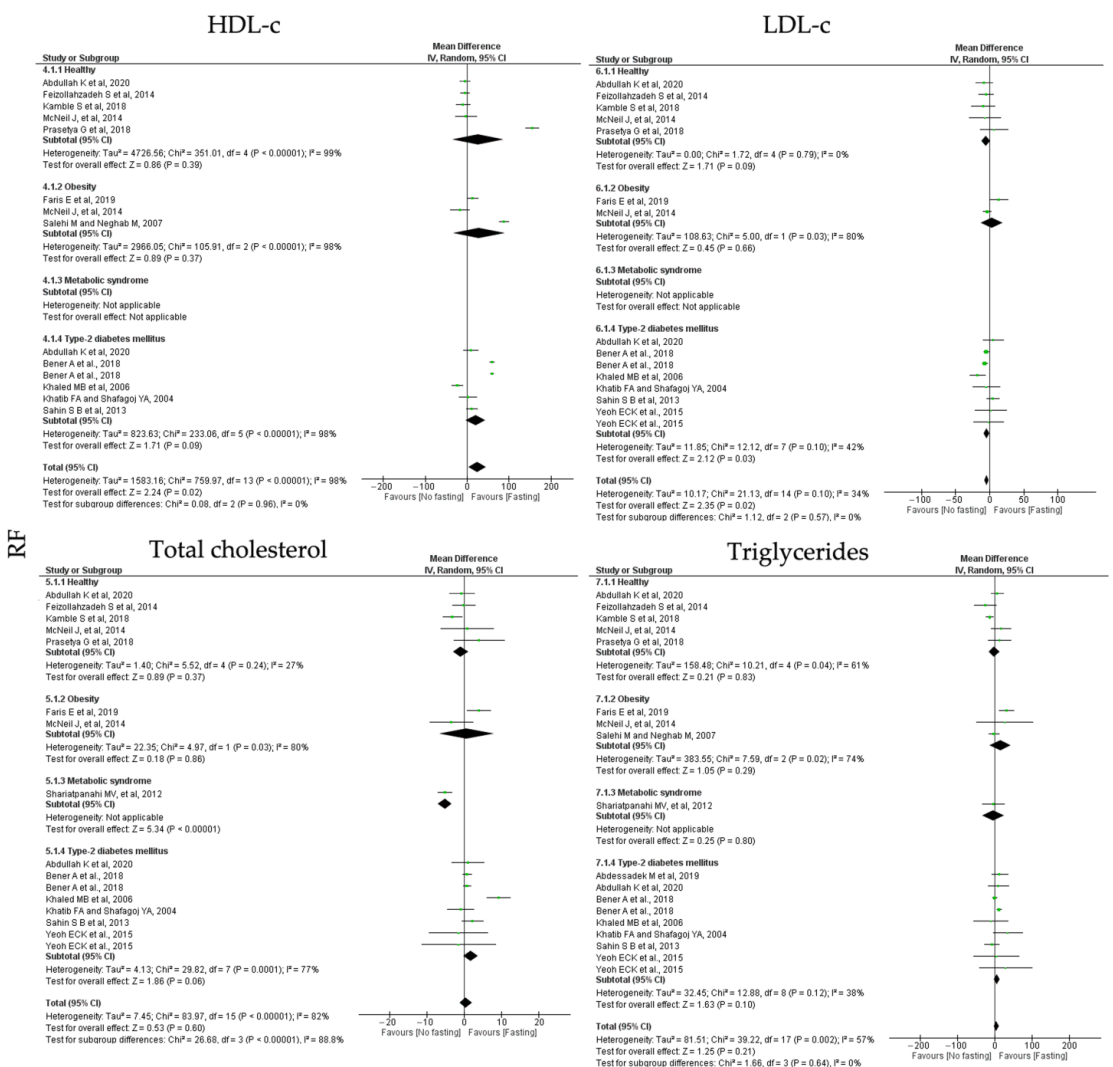
Forest plot of the data from random effects meta-analysis shown as mean difference with 95% confidence intervals on lipid homeostasis outcomes, HDL-c, LDL-c, total cholesterol, triglycerides, for the studies that presented data concerning these parameters. Data are presented according to religious fasting (RF) and the metabolic conditions: healthy or disease condition, obesity, T2D or MetS. For each study, the square represents the mean difference between baseline and fasting conditions, with the horizontal line intersecting it as the lower and upper limits of the 95% confidence interval [17,18,21,28,36,37,39,40,45,48,49,50,57].

To study the impact of the different types of IF on lipid homeostasis, the outcomes analyzed were HDL-c, LDL-c, total cholesterol and triglyceride. ADF, TRF and RF showed distinct impact on the analyzed lipid homeostasis outcomes (Table 3, Table 4 and Table 5; Figure 5, Figure 6 and Figure 7). Starting with the **ADF** protocols, data collected revealed that this intervention promotes statistically significant positive effects on HDL-c (k = 11, 1.49 (95% CI [0.72, 2.26]), *p* = 0.0002), LDL-c (k = 12, 7.06 (95% CI [3.19, 10.93]), *p* = 0.0004) and total cholesterol (k = 12, 13.18 (95% CI [9.16, 17.16]), *p* < 0.00001) levels. Heterogeneity between studies, related with each outcome, was high (Table 3; Figure 5).

Regarding HDL-c, LDL-c and total cholesterol parameters, only people with obesity presented statistically significant positive effects (k = 8, 1.59 (95% CI [0.79, 2.39]), *p* < 0.00001; (k = 8, 7.67 (95% CI [3.51, 11.83], *p* = 0.0003 and k = 8, 13.87 (95% CI [9.62, 18.12, *p* < 0.00001, respectively) (Table 3). High heterogeneity was observed in studies involving obese group (Q = 796.57, *p* < 0.00001, I2 = 99.00%; Q = 22,238.46, *p* < 0.00001, I2 = 100.00% and Q = 22,062.59, *p* < 0.00001, I2 = 100.00%, respectively) (Table 3; Figure 5).

Finally, ADF protocols also significantly decreased triglycerides levels in MetS participants (k = 1, 52.00 (95% CI [9.41, 94.59]), *p* = 0.02) (Table 3; Figure 5).

**Table 4:** Analysis of the impact of Time-Restricted Fasting (TRF) protocols on different outcomes in healthy individuals and/or individuals with metabolic related disorders as type 2 diabetes *mellitus* (T2D), metabolic syndrome (MetS) or obesity.

	Moderators	k ^1^	Point Estimate	CI Lower	CI Upper	*p*-Value	Heterogeneity		
							Q-Value	*p*-Value	I-Squared
**Adiposity**	Weight (kg)								
	All studies	13	3.05	0.87	5.23	0.006	46.60	<0.00001	72.00
	Healthy	1	1.04	−1.89	3.97	0.49	-	-	-
	Obesity	7	2.78	1.50	4.06	<0.00001	1.37	0.99	0.00
	T2D	2	0.89	−6.55	8.34	0.81	0.00	0.95	0.00
	MetS	3	7.14	3.71	10.57	<0.00001	2.46	0.29	19.00
	BMI (kg/m^2^)								
	All studies	9	1.48	0.39	2.57	0.008	45.32	<0.0001	82.00
	Healthy	0	-	-	-	-	-	-	-
	Obesity	5	1.01	0.56	1.46	<0.00001	2.97	0.56	0.00
	T2D	1	3.50	−3.69	10.69	0.34	-	-	-
	MetS	3	2.01	0.24	3.78	0.03	4.92	0.09	59.00
	Waist circumference (cm)								
	All studies	8	3.93	2.64	5.21	<0.00001	0.98	1.00	0.00
	Healthy	0	-	-	-	-	-	-	-
	Obesity	5	3.89	2.54	5.23	<0.00001	0.57	0.97	0.00
	T2D	1	1.80	−7.93	11.53	0.72	-	-	-
	MetS	2	5.01	0.12	9.90	0.04	0.03	0.87	0.00
**Lipid homeostasis**	HDL-c (mg/dL)								
	All studies	8	0.00	−0.60	0.61	0.99	8.96	0.44	0.00
	Healthy	0	-	-	-	-	-	-	-
	Obesity	5	0.43	−0.46	1.31	0.34	5.08	0.41	2.00
	T2D	1	0.90	−3.36	5.16	0.72	-	-	-
	MetS	3	−0.44	−1.29	0.42	0.32	1.82	0.40	0.00
	LDL-c (mg/dL)								
	All studies	7	4.48	−7.60	16.56	0.47	200.73	<0.00001	97.00
	Healthy	0	-	-	-	-	-	-	-
	Obesity	3	−1.47	−7.32	4.38	0.62	15.32	0.002	80.00
	T2D	1	3.87	−17.06	24.80	0.72	-	-	-
	MetS	3	14.16	−7.92	36.28	0.21	12.49	0.002	84.00
	Total cholesterol (mg/dL)								
	All studies	8	8.14	−8.37	24.65	0.33	154.28	<0.00001	95.00
	Healthy	0	-	-	-	-	-	-	-
	Obesity	4	−4.21	−10.36	1.94	0.18	4.75	0.19	37.00
	T2D	1	3.80	−17.13	24.73	0.72	-	-	-
	MetS	3	28.80	25.26	32.34	<0.00001	1.98	0.37	0.00
	Triglycerides (mg/dL)								
	All studies	9	8.93	−1.58	19.45	0.10	80.70	<0.00001	89.00
	Healthy	1	7.08	−2.08	16.24	0.13	-	-	-
	Obesity	4	3.41	−2.80	9.63	0.28	10.66	0.03	62.00
	T2D	1	0.00	−42.34	42.34	1.00	-	-	-
	MetS	3	20.61	−12.67	53.90	0.22	6.59	0.04	70.00
**Insulin homeostasis**	Fasting glucose (mg/dL)								
	All studies	10	5.89	2.52	9.26	0.0006	338.62	<0.00001	97.00
	Healthy	1	0.54	−1.33	2.41	0.57	-	-	-
	Obesity	5	4.25	0.32	8.18	0.03	102.00	<0.00001	95.00
	T2D	2	7.22	3.74	10.71	<0.00001	13.45	0.0002	93.00
	MetS	2	13.75	7.99	19.50	<0.00001	1.73	0.19	42.00
	Fasting insulin (mU/L)								
	All studies	9	2.34	0.17	4.51	0.003	24.85	0.0002	68.00
	Healthy	1	1.58	−8.05	11.21	0.75	-	-	-
	Obesity	5	1.19	0.14	2.23	0.03	18.09	0.0001	78.00
	T2D	1	−2.70	−15.93	10.53	0.69	-	-	-
	MetS	2	2.27	1.04	3.51	0.0003	0.15	0.70	0.00
	HOMA-IR								
	All studies	5	0.75	0.35	1.15	0.0002	15.54	0.0008	68.00
	Healthy	0	-	-	-	-	-	-	-
	Obesity	2	0.57	0.21	0.93	0.002	4.14	0.13	52.00
	T2D	1	0.40	−1.98	2.78	0.74	-	-	-
	MetS	2	1.29	0.99	1.60	<0.00001	0.03	0.86	0.00
**Blood pressure**	SBP (mmHg)								
	All studies	11	3.99	1.64	6.34	0.0009	57.42	<0.00001	81.00
	Healthy	0	-	-	-	-	-	-	-
	Obesity	7	3.18	0.29	6.07	0.03	38.68	<0.00001	82.00
	T2D	2	4.77	−1.11	10.64	0.11	0.05	0.83	0.00
	MetS	2	7.27	5.91	8.63	<0.00001	0.35	0.55	0.00
	DBP (mmHg)								
	All studies	11	2.86	1.78	3.94	<0.00001	38.22	<0.00001	71.00
	Healthy	0	-	-	-	-	-	-	-
	Obesity	7	2.36	1.47	3.24	<0.00001	11.78	0.11	41.00
	T2D	2	3.80	0.66	6.94	0.02	0.24	0.62	0.00
	MetS	2	4.77	4.15	5.38	<0.00001	0.29	0.59	0.00

^1^ k—number of studies; MetS—Metabolic syndrome; T2D—diabetes *mellitus* type 2; HOMA—insulin resistance; BMI—Body mass index; SBP—Systolic Blood Pressure; DBP—Diastolic Blood Pressure.

For **TRF** protocols, the analyzed data revealed distinct results among the lipid homeostasis outcomes. TRF interventions showed null effects for HDL-c (k = 8, 0.00 (95% CI [−0.60, 0.61]), *p* = 0.99), and a positive trend to reduce the LDL-c (k = 7, 4.48 (95% CI [−7.60, 16.56]), *p* = 0.47), total cholesterol (k = 8, 8.14 (95% CI [−8.37, 24.65]), *p* = 0.33) and triglycerides (k = 9, 8.93 (95% CI [−1.58, 19.45]), *p* = 0.10) levels (Table 3; Figure 3). With exception between the HDL-c studies, the heterogeneity between the studies compiling the data for LDL-c, total cholesterol and triglycerides was high (Table 4; Figure 6).

Individual analysis revealed the only MetS individuals displayed statistically significant decreased levels of total cholesterol after TRF intervention (k = 3, 28.80 (95% CI [25.26, 32.34, *p* < 0.00001). Low heterogeneity was observed between the studies for the MetS group (Q = 1.98, *p* = 0.37, I2 = 0.00%) (Table 4; Figure 6).

**Table 5:** Analysis of the impact of religious protocols, namely Ramadan, on different outcomes in healthy individuals and/or individuals with metabolic related disorders as type 2 diabetes *mellitus* (T2D), metabolic syndrome (MetS) or obesity.

	Moderators	k ^1^	Point Estimate	CI Lower	CI Upper	*p*-Value	Heterogeneity		
							Q-Value	*p*-Value	I-Squared
**Adiposity**	Weight (kg)								
	All studies	14	2.22	1.07	3.38	0.0002	8.11	0.80	0.00
	Healthy	5	2.44	0.74	4.14	0.005	3.84	0.43	0.00
	Obesity	6	3.01	0.92	5.10	0.005	1.95	0.75	0.00
	T2D	4	0.78	−1.60	3.15	0.52	0.29	0.99	0.00
	MetS	0	-	-	-		-	-	-
	BMI (kg/m^2^)								
	All studies	19	1.01	0.81	1.21	<0.00001	18.42	0.73	0.00
	Healthy	5	0.70	0.32	1.09	0.0004	2.46	0.65	0.00
	Obesity	7	0.84	0.43	1.26	<0.00001	2.57	0.86	0.00
	T2D	8	1.26	0.98	1.54	<0.00001	7.23	0.70	0.00
	MetS	1	0.75	−0.67	2.17	<0.00001	-	-	-
	Waist circumference (cm)								
	All studies	6	1.66	0.18	3.15	0.03	2.09	0.95	0.00
	Healthy	3	1.32	−1.35	3.99	0.33	1.04	0.59	0.00
	Obesity	2	1.30	−2.84	5.44	0.54	0.09	0.77	0.00
	T2D	2	0.76	−2.54	4.07	0.65	0.03	0.87	0.00
	MetS	1	2.61	0.12	5.10	0.04	-	-	-
**Lipid homeostasis**	HDL-c (mg/dL)								
	All studies	12	0.44	−1.21	2.10	0.60	83.97	<0.00001	82.00
	Healthy	5	−0.91	−2.90	1.08	0.37	5.52	0.24	27.00
	Obesity	2	0.67	−6.61	7.96	0.86	4.97	0.03	80.00
	T2D	6	1.77	−0.10	3.64	0.06	29.82	<0.00001	77.00
	MetS	1	−5.13	−7.01	3.25	<0.00001	-	-	-
	LDL-c (mg/dL)								
	All studies	11	−3.81	−6.99	−0.63	0.02	21.13	0.10	34.00
	Healthy	5	−5.68	−12.21	0.85	0.09	1.72	0.79	0.00
	Obesity	2	3.64	−12.36	19.64	0.66	5.00	0.03	80.00
	T2D	6	−4.55	−8.75	−0.35	0.03	12.12	0.10	42.00
	MetS	0	-	-	-	-	-	-	-
	Total cholesterol (mg/dL)								
	All studies	11	24.29	3.05	45.53	0.02	759.97	<0.00001	98.00
	Healthy	5	26.54	−34.18	87.26	0.39	351.01	<0.00001	99.00
	Obesity	3	28.36	−33.99	90.70	0.37	105.91	<0.00001	98.00
	T2D	5	20.58	−3.03	44.19	0.09	233.06	<0.00001	98.00
	MetS	0	-	-	-				
	Triglycerides (mg/dL)								
	All studies	14	4.31	−2.44	11.07	0.21	39.22	0.002	57.00
	Healthy	5	−1.61	−16.33	13.11	0.83	10.21	0.04	61.00
	Obesity	3	14.96	−12.98	42.89	0.29	7.59	0.02	74.00
	T2D	7	6.06	−1.24	13.35	0.10	12.88	0.12	38.00
	MetS	1	−3.78	−33.78	26.22	0.80	-	-	-
**Insulin homeostasis**	Fasting glucose (mg/dL)								
	All studies	13	13.75	2.47	25.02	0.02	1360.6	<0.00001	99.00
	Healthy	3	−3.65	−8.24	0.95	0.12	7.85	0.02	75.00
	Obesity	4	1.06	−5.23	7.34	0.74	7.71	0.05	61.00
	T2D	6	29.90	21.29	38.50	<0.00001	64.87	<0.00001	91.00
	MetS	1	20.24	12.38	28.10	<0.00001	-	-	-
	Fasting insulin (mU/L)								
	All studies	7	−1.06	−3.17	1.06	0.33	39.87	<0.00001	80.00
	Healthy	4	0.05	−1.24	1.34	0.94	3.64	0.30	18.00
	Obesity	3	−3.20	−7.14	0.74	0.11	5.18	0.07	61.00
	T2D	2	−1.07	−8.28	6.13	0.77	5.52	0.02	82.00
	MetS	0	-	-	-	-	-	-	-
	HOMA-IR								
	All studies	6	0.32	−0.91	1.54	0.64	448.84	<0.00001	98.00
	Healthy	4	−0.05	−0.26	0.16	0.63	3.61	0.31	17.00
	Obesity	3	1.49	−1.68	4.67	0.36	202.77	<0.00001	99.00
	T2D	1	−0.68	−1.51	0.15	0.11	-	-	-
	MetS	0	-	-	-	-	-	-	-
**Blood pressure**	SBP (mmHg)								
	All studies	7	1.54	−1.29	4.37	0.29	79.27	<0.00001	89.00
	Healthy	1	−0.22	−1.92	1.48	0.80	-	-	-
	Obesity	3	2.00	−1.19	5.19	0.22	4.14	0.13	52.00
	T2D	4	1.84	−3.11	6.78	0.47	71.50	<0.00001	93.00
	MetS	0	-	-	-	-	-	-	-
	DBP (mmHg)								
	All studies	7	0.51	−0.38	1.40	0.27	16.33	0.06	45.00
	Healthy	1	−0.67	−2.42	1.08	0.45	-	-	-
	Obesity	3	0.92	−1.12	2.96	0.38	0.59	0.74	0.00
	T2D	4	0.65	−0.58	1.88	0.30	13.59	0.02	63.00
	MetS	0	-	-	-	-	-	-	-

^1^ k—number of studies; MetS—Metabolic syndrome; T2D—diabetes *mellitus* type 2; HOMA—insulin resistance; BMI—Body mass index; SBP—Systolic Blood Pressure; DBP—Diastolic Blood Pressure.

**RF** protocols promoted a non-significant increase in HDL-c (k = 12, 0.44 (95% CI [−1.21, 2.10]), *p* = 0.60) and triglycerides levels (k = 14, 4.31 (95% CI [−2.44, 11.07]), *p* = 0.21); a significant reduction in total cholesterol (k = 11, 24.29 (95% CI [3.05, 45.53]), *p* = 0.02) and a negative significant increase in LDL-c levels (k = 11, −3.81 (95% CI [−6.99, −0.63]), *p* = 0.02) (Table 5). Heterogeneity between the studies for LDL-c and total cholesterol was high (Table 4) and it was moderate between the studies presenting data for LDL-c and triglycerides levels (Table 5; Figure 7).

MetS individuals are negatively affected by RF intervention, with a statistically significance decrease in HDL-c levels (k = 1, −5.13 (95% CI [−7.01, 3.25]), *p* < 0.00001) (Table 5). Whereas, for T2D individuals, RT protocols increased LDL-c levels, (k = 6, −4.55 (95% CI [−8.75, −0.35], *p* = 0.03), with moderate heterogeneity between the studies (Q = 12.12, *p* = 0.10, I2 = 42.00) (Table 5; Figure 7).

#### 3.6.3. Insulin Homeostasis

**Figure 8:**
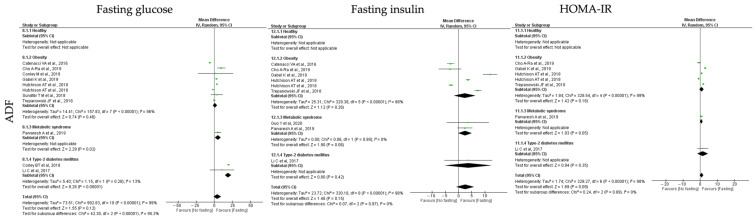
Forest plot of the data from random effects meta-analysis shown as mean difference with 95% confidence intervals on insulin homeostasis outcomes, fasting glucose, fasting insulin, insulin resistance (HOMA-IR), for the studies that presented data concerning these parameters. Data are presented according to alternate-day fasting (ADF) and the metabolic conditions: healthy or disease condition, obesity, T2D or MetS. For each study, the square represents the mean difference between baseline and fasting conditions, with the horizontal line intersecting it as the lower and upper limits of the 95% confidence interval [9,22,23,25,29,32,34,43,47,54,61].

Regarding insulin homeostasis, the parameters analyzed were fasting glucose, fasting insulin and HOMA-IR levels. In relation to the impact of **ADF** interventions on the outcomes of insulin homeostasis, global data analysis revealed that ADF protocols showed a non-significant reduction in fasting glucose, fasting insulin and HOMA-IR levels (k = 10, 4.33 (95% CI [−1.14, 9.79]), *p* = 0.12; k = 8, 2.55 (95% CI [−0.88, 5.98]), *p* = 0.15 and k = 6, 0.90 (95% CI [−0.14, 1.95]), *p* = 0.09, respectively). High heterogeneity was detected between the studies (Table 3; Figure 8).

Group stratification revealed that ADF protocols promoted a significant reduction in fasting glucose levels in T2D and MetS participants (k = 2, 19.17 (95% CI [14.68, 23.72]), *p* < 0.00001 and k = 1, 5.00 (95% CI [0.71, 9.29]), *p* = 0.02, respectively). Studies comprising T2D participants have low heterogeneity (Q = 1.15, *p* = 0.28, I2 = 13.00%) (Table 3; Figure 4). Furthermore, HOMA-IR levels were statistically significant reduced in MetS individuals after ADF protocols (k = 1, 0.73 (95% CI [−0.01, 1.47, *p* < 0.00001) (Table 3; Figure 8).

**Figure 9:**
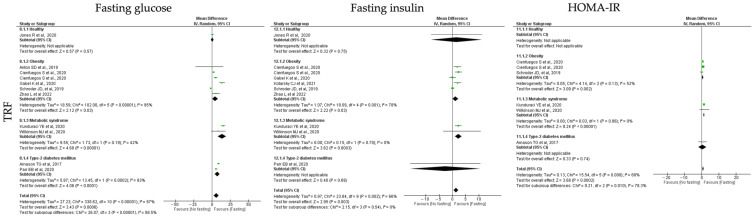
Forest plot of the data from random effects meta-analysis shown as mean difference with 95% confidence intervals on insulin homeostasis outcomes, fasting glucose, fasting insulin, insulin resistance (HOMA-IR), for the studies that presented data concerning these parameters. Data are presented according to time-restricted fasting (TRF) and the metabolic conditions: healthy or disease condition, obesity, T2D or MetS. For each study, the square represents the mean difference between baseline and fasting conditions, with the horizontal line intersecting it as the lower and upper limits of the 95% confidence interval [7,19,20,24,30,35,46,51,56,58].

For the **TRF** protocols, data revealed a significant reduction in fasting glucose, fasting insulin and HOMA-IR levels (k = 10, 5.89 (95% CI [2.52, 9.26]), *p* = 0.0006; k = 9, 2.34 (95% CI [0.17, 4.51]), *p* = 0.003 and k = 5, 0.75 (95% CI [0.35, 1.15]), *p* = 0.0002, respectively). High heterogeneity was observed between the studies (Table 4; Figure 9).

Fasting glucose levels were statistically significant reduced by TRF protocols in obese, T2D and MetS individuals (k = 5, 4.25 (95% CI [0.32, 8.18]), *p* = 0.03; k = 2, 7.22 (95% CI [3.74, 10.71]), *p* < 0.00001 and k = 2, 13.75 (95% CI [7.99, 19.50]), *p* < 0.00001, respectively). Heterogeneity between the studies was high for obese and T2D individuals (Q = 102.00, *p* < 0.00001, I2 = 95.00% and Q = 13.45, *p* = 0.0002, I2 = 93.00%, respectively), and moderate for MetS individuals (Q = 1.73, *p* = 0.19, I2 = 42.00%) (Table 4; Figure 9).

Concerning the fasting insulin levels, they displayed a statistically significant reduction in obese and MetS individuals (k = 5, 1.19 (95% CI [0.14, 2.23]), *p* = 0.03 and k = 2, 2.27 (95% CI [1.04, 3.51]), *p* = 0.0003, respectively). Low heterogeneity was observed between the studies for the MetS group (Q = 0.15, *p* = 0.70, I2 = 0.00%), while moderate heterogeneity was detected for the obesity group (Q = 18.09, *p* = 0.0001, I2 = 78.00%) (Table 4; Figure 9).

Finally, TRF protocols promoted a statistically significant reduction in HOMA-IR levels in obese and MetS individuals (k = 2, 0.57 (95% CI [0.21, 0.93]), *p* = 0.002 and k = 2, 1.29 (95% CI [0.99, 1.60]), *p* < 0.00001, respectively). Heterogeneity was moderate in studies with obesity (Q = 4.14, *p* = 0.13, I2 = 52.00%) and low for the studies with MetS participants (Q = 0.03, *p* = 0.86, I2 = 0.00%) groups (Table 4; Figure 9).

**Figure 10:**
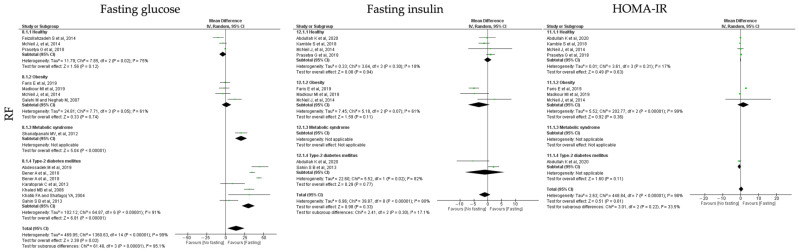
Forest plot of the data from random effects meta-analysis shown as mean difference with 95% confidence intervals on insulin homeostasis outcomes, fasting glucose, fasting insulin, insulin resistance (HOMA-IR), for the studies that presented data concerning these parameters. Data are presented according to religious fasting (RF) and the metabolic conditions: healthy or disease condition, obesity, T2D or MetS. For each study, the square represents the mean difference between baseline and fasting conditions, with the horizontal line intersecting it as the lower and upper limits of the 95% confidence interval [17,18,21,27,28,37,39,40,44,45,48,49,50,53].

In relation to the **RF** protocols, data showed that these interventions promote a statistically significant reduction in fasting glucose (k = 13, 13.75 (95% CI [2.47, 25.02]), *p* = 0.02), a non-significant alteration of HOMA-IR levels (k = 6, 0.32 (95% CI [−0.91, 1.54]), *p* = 0.64) and a slight increase in fasting insulin (k = 7, −1.06 (95% CI [−3.17, 1.06]), *p* = 0.33) (Table 4). Heterogeneity between the studies was high (Table 5; Figure 10).

The stratification by groups showed that RF protocols only promoted a significant reduction in fasting glucose levels in T2D and MetS individuals (k = 6, 29.90 (95% CI [21.29, 38.50]), *p* < 0.00001 and k = 1, 20.24 (95% CI [12.38, 28.10]), *p* < 0.00001, respectively). T2D group showed high heterogeneity between the selected studies (Q = 64.87, *p* < 0.00001, I2 = 91.00%) (Table 5; Figure 10).

#### 3.6.4. Blood Pressure

**Figure 11:**
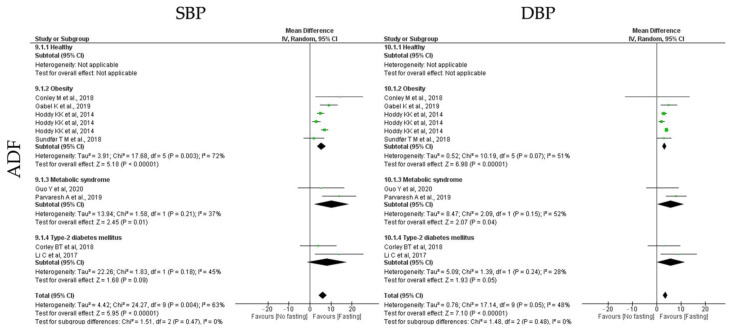
Forest plot of the data from random effects meta-analysis shown as mean difference with 95% confidence intervals on blood pressure outcomes systolic blood pressure (SBP) and diastolic blood pressure (DBP), for the studies that presented data concerning these parameters. Data are presented according to alternate-day fasting (ADF) and the metabolic conditions: healthy or disease condition, obesity, T2D or MetS. For each study, the square represents the mean difference between baseline and fasting conditions, with the horizontal line intersecting it as the lower and upper limits of the 95% confidence interval [9,25,29,32,33,43,47,54].

**Figure 12:**
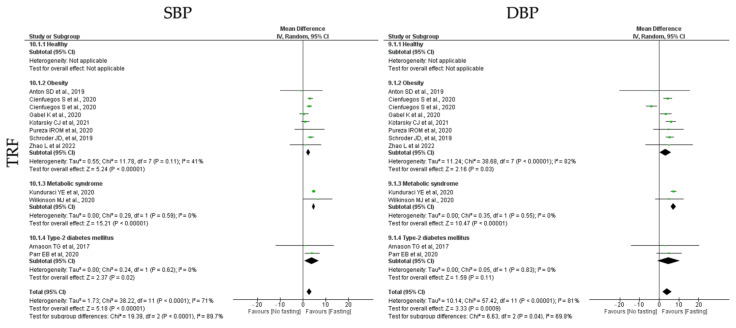
Forest plot of the data from random effects meta-analysis shown as mean difference with 95% confidence intervals on blood pressure outcomes systolic blood pressure (SBP) and diastolic blood pressure (DBP), for the studies that presented data concerning these parameters. Data are presented according to time-restricted fasting (TRF) and the metabolic conditions: healthy or disease condition, obesity, T2D or MetS. For each study, the square represents the mean difference between baseline and fasting conditions, with the horizontal line intersecting it as the lower and upper limits of the 95% confidence interval [7,19,20,24,26,30,41,46,51,56,58].

**Figure 13:**
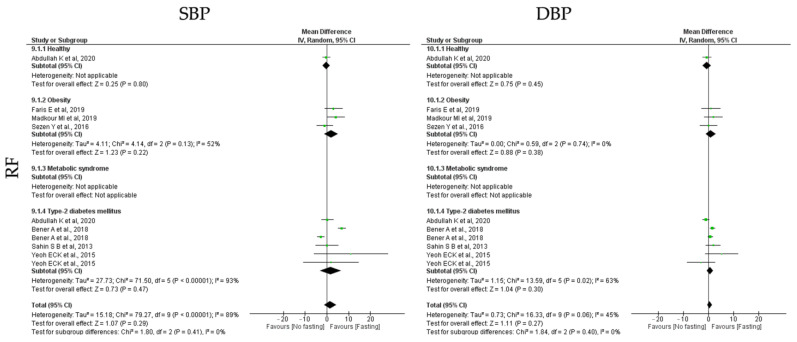
Forest plot of the data from random effects meta-analysis shown as mean difference with 95% confidence intervals on blood pressure outcomes systolic blood pressure (SBP) and diastolic blood pressure (DBP), for the studies that presented data concerning these parameters. Data are presented according to religious fasting (RF) and the metabolic conditions: healthy or disease condition, obesity, T2D or MetS. For each study, the square represents the mean difference between baseline and fasting conditions, with the horizontal line intersecting it as the lower and upper limits of the 95% confidence interval [18,21,27,44,49,52,57].

To study the impact of different types of IF protocols on blood pressure, the outcomes systolic blood pressure (SBP) and diastolic blood pressure (DBP) were analyzed. Relatively to blood pressure outcomes, ADF, TRF and RF showed positive impact (Table 3, Table 4 and Table 5 and Figure 11, Figure 12 and Figure 13).

**ADF** protocols promoted a statistically significant reduction in SBP (k = 8, 6.08 (95% CI [4.08, 8.08]), *p* < 0.00001) and DBP (k = 8, 3.52 (95% CI [2.55, 4.49]), *p* < 0.00001) (Table 3). For each outcome, the heterogeneity between studies was moderate (Table 3; Figure 11).

SBP was statistically significant reduced in obese and MetS individuals (k = 4, 5.49 (95% CI [3.41, 7.57]), *p* < 0.00001 and k = 2, 10.46 (95% CI [2.08, 18.83]), *p* = 0.01, respectively), after ADF protocols. Heterogeneity between the studies for obesity and MetS groups was moderate (Q = 17.68, *p* = 0.003, I2 = 72.00% and Q = 1.58, *p* = 0.21, I2 = 37.00%, respectively). In relation to DBP, a statistically significant reduction was observed for obese, T2D and MetS individuals (k = 4, 3.23 (95% CI [2.32, 4.13]), *p* < 0.00001, k = 2, 5.79 (95% CI [−0.08, 11.65]), *p* = 0.05 and k = 2, 5.76 (95% CI [0.30, 11.22]), *p* = 0.04, respectively), with moderate heterogeneity between studies (Q = 10.19, *p* = 0.07, I2 = 51.00%, Q = 1.39, *p* = 0.24, I2 = 28.00% and Q = 2.09, *p* = 0.15, I2 = 52.00%, respectively) (Table 3; Figure 11).

**TRF** protocols also promoted a statistically significant positive reduction in both SBP and DBP (k = 11, 3.99 (95% CI [1.64, 6.34]), *p* = 0.0009 and k = 11, 2.86 (95% CI [1.47, 3.24]), *p* < 0.00001, respectively), with high and moderate studies heterogeneity, respectively (Q = 57.42, *p* < 0.00001, I2 = 81.00% and Q = 38.22, *p* < 0.00001, I2 = 71.00%, respectively) (Table 4; Figure 12).

A statistically significant reduction in SBP was observed in people with obesity and MetS individuals (k = 7, 3.18 (95% CI [0.29, 6.07]), *p* = 0.03 and k = 2, 7.27 (95% CI [5.91, 8.63]), *p* < 0.00001, respectively), after TRF protocols. Heterogeneity between the studies was high for the obesity group (Q = 38.68, *p* < 0.00001, I2 = 82.00%) and low for MetS groups (Q = 0.35, *p* = 0.55, I2 = 0.00%). Concerning DBP, a statistically significant reduction was observed in obese, T2D and MetS individuals (k = 4, 3.23 (95% CI [2.32, 4.13]), *p* < 0.00001, k = 2, 5.79 (95% CI [−0.08, 11.65]), *p* = 0.05 and k = 2, 5.76 (95% CI [0.30, 11.22]), *p* = 0.04, respectively), after TRF protocols. Heterogeneity was moderate for obese group and low for T2D and MetS groups (Q = 11.78, *p* = 0.11, I2 = 41.00%, Q = 0.24, *p* = 0.62, I2= 0.00% and Q = 0.29, *p* = 0.59, I2 = 0.00%, respectively) (Table 4; Figure 12).

Finally, **RF** protocols only promoted no significant impact of SBP and DBP (k = 7, 1.54 (95% CI [−1.29, 4.37]), *p* = 0.29 and k = 7, 0.51 (95% CI [−0.38, 1.40]), *p* = 0.27, respectively), with high and moderate heterogeneity between studies, respectively (Q = 79.27, *p* < 0.00001, I2 = 89.00% and Q = 16.33, *p* = 0.06, I2 = 45.00, respectively) (Table 5; Figure 13).

This meta-analysis demonstrates high heterogeneity in the results, particularly in studies of individuals with obesity and MetS (Table 3, Table 4 and Table 5). Therefore, a sensitive analysis was performed by two different strategies. In the first, the sensitive analysis integrated all the studies (Table S3), and in the second analysis the impact of IF, without the RF studies was evaluated (Table S4). The sensitivity analysis did not decrease heterogeneity neither when all studies are integrated nor when RF studies were removed. Therefore, data suggest that heterogeneity is most probably related with differences in study design, populations, interventions or outcomes across the included studies.

## 4. Discussion

The main purpose of this systematic review and meta-analysis was to summarize scientific evidence on the impact of IF protocols on metabolic-related outcomes, both on healthy and metabolic-related disease conditions, namely obesity, T2D and MetS. To our knowledge, this is the first meta-analysis that presents comprehensive results on the effects of different IF protocols, Alternate-Day Fasting (ADF), Time-Restricted Fasting (TRF) and Religious Fasting (RF), on specific parameters both in health and in metabolic-related disorders. Despite the amount of data already available and the fact that many studies point to beneficial effects of IF protocols on several metabolic parameters, data are conflicting. While reasons are still elusive, we hypothesized that different basal metabolic conditions such as those herein analyzed, as well as the multitude of IF protocols could contribute for distinct results and conclusions.

Herein, we have considered not only the effectiveness of different IF protocols but also how they impact in individuals with different metabolic status. For our analysis we selected three main common metabolic disorders: obesity, T2D and MetS. Regarding each of the metabolic parameters, for the adiposity outcomes, it was possible to observe that the different IF approaches resulted in the reduction in weight, BMI and waist circumference. These data suggest that independently of the IF intervention protocol, and most probably the reasons behind these observations are most likely related with the inability of individuals to fully compensate, during non-fasting periods, the calorie deficit associated with IF protocols [76,77,78]. Furthermore, fasting periods might also diminish the hunger that these individuals usually feel [77,79,80,81,82]. For the remaining outcomes, namely lipid, insulin and blood pressure homeostasis, ADF was the type of IF that showed the most beneficial effects in comparison with TRF and RF. The ADF major effectiveness might be related with higher fasting time of this intervention and a greater overall caloric restriction. These alterations can elicit an augmented autophagy, improved insulin sensitivity and reduce inflammation, possibly by modulating the gut microbiota and the release of inflammatory cytokines [3,83,84]. Furthermore, the increment of fasting periods contributes to a decreased in energy intake [85], to a depletion of liver glycogen and a metabolic switch from lipid/cholesterol synthesis and fat storage to the utilization of fat as a substrate [86]. Therefore, IF protocols’ effectiveness might be depend on different factors such as the duration and frequency of fasting periods, the amount and type of food consumed during feeding periods, and individual variation in genetic and metabolic factors [3,22,87].

Although RF have a similar fasting time, when compared to TRF, this last appears to present more pronounced beneficial effects. The differences between TRF and RF are most probably associated with the alterations promoted by RF in the circadian rhythm. It has been postulated that TRF, due to the limited eating windows, allow for the synchronization of the circadian system, consequently optimizing the metabolic function [88]. Indeed, it was already described that both TRF and ADF have beneficial effects aligned with the circadian rhythm, which have consequently benefits on glucose regulation, beta cell responsiveness, body composition and weight, reduction in oxidative stress and metabolic switch [3,83,84,89]. RF disturbs the circadian system, which, as previously described, can predispose to several dysfunctions, such as the impairment of glucose tolerance, reduction in sensitivity to insulin and increased arterial blood pressure [3,83,84,89].

Our analysis shows that a significant weight loss, reduction in BMI and waist circumference is particularly observed in obese and MetS individuals independently of IF approach. MetS constitutes a group with a small number of studies that did not provide enough information to allow a categorization of the participants in either people with obesity or T2D. However, one has to keep in mind that MetS is a combination of several metabolic disfunctions, including obesity, (one of the metabolic disorders herein analyzed) insulin resistance, hypertriglyceridemia, hypercholesterolemia, hypertension and reduced high-density lipoprotein (HDL)-cholesterol concentrations [90]. Therefore, the promoted metabolic shift and the reduction in fat mass, induced by the IF protocols, could underlie the beneficial effects observed in the lipid profile of obese and MetS individuals, in regards to the increment of HDL-c and the reduction in the total cholesterol and triglycerides, as already reported in the literature (reviewed in [91]). T2D individuals benefit by the implementation of IF protocols through the reduction in fasting glucose and insulin. T2D is triggered by a combination of two essential factors, a defect in insulin secretion and/or the inability of insulin-sensitive tissues to respond appropriately to insulin [90]. It is proposed that these insulin defects are linked to increased adiposity and subsequent chronic inflammation, leading to the development of insulin resistance in tissues [90]. Therefore, we hypothesized that reduction in caloric intake and the consequent metabolic changes, implied by IF, underly the establishment of a better insulin homeostasis. Furthermore, it is also described that IF protocols might incite a prolonged decrease in insulin production and increased levels of AMPK, which can result in the improvement of insulin sensitivity and glucose homeostasis [92]. In relation to healthy individuals, data herein presented have no power to infer the role of IF in the modulation of the metabolic parameters analyzed, under healthy circumstances. Cohorts of healthy individuals are reduced and essentially subjected to RF, which presented the worst IF results in health promotion.

This work faced several limitations including great heterogeneity between studies, lack of blinding and variable quality of result reporting. The high heterogeneity observed in the global analysis can be explained by the strategy of grouping participants with different metabolic status, the diversity of IF interventions, variation in population age, gender ratio and geographical localization. In addition, individual weight loss and behavior change, such as increased physical activity or improved dietary habits, can be confounding factors when assessing the benefits of IF. Weight loss, a common outcome of IF, can, independently of IF intervention, lead to improvements in metabolic markers, such as insulin sensitivity and lipid profiles, making it difficult to determine which factor is responsible for the enhancement of metabolic health [93,94]. Therefore, it is important to control for these factors when designing and conducting studies on the effects of IF. However, the observed heterogeneity, by other side, can be seen as a major find, suggesting that IF should be personalized. Other aspect is that the literature search ranged from 2000 to June 2022. Although the concept of IF and its variations have been around for centuries, and various cultures and religions have practiced fasting for spiritual, health or cultural reasons, the scientific understanding and interest in IF gained more attention and recognition in the scientific community in the 21st century. By the year 2000, the term IF was widely used in the scientific literature to describe a variety of different fasting protocols, including ADF, TRF and RF. Furthermore, it is only in the last few decades that IF protocols have become more standardized. Therefore, for a more consistent classification, we decided to select studies published after the year 2000, keeping in mind that this is one of the study limitations.

Another point to consider in this study is that the commonly used measures of IF, such as changes in body weight, blood glucose levels or lipid profiles, may not be perfect proxies to estimate the metabolic effects of IF. Indeed, these measures can be easily influenced by other factors, for example body weight, changes in water weight, muscle mass and measurements under fasting or feeding conditions, rather than just changes in fat mass [93,95]. Importantly, there are other measures that may provide more accurate insights into the cellular mechanisms underlying the metabolic IF benefits. Examples include changes in biomarkers of cellular stress, oxidative damage or even autophagy [81,83,96]. Nevertheless, the measurement of these biomarkers is not well defined in the clinical context, neither included in the hospitals’ routine analysis. Therefore, new approaches must be applied in order to overcome this drawback.

Despite the described limitations, one of the main findings of this study is that IF interventions have beneficial effects for most of individuals included in the studies, and independently of the IF protocol used. IF has been associated with improvements in weight loss, control of blood sugar and blood pressure and cholesterol levels [3,75,88,97,98,99]. However, not all individuals may benefit from IF, since it is described that some individuals may experience negative side effects such as hunger, fatigue and irritability [84,99]. IF benefits are mostly dependent of mechanisms associated with a metabolic shift towards the predominant use of fatty acids as fuel for energy [3,100]. However, IF benefits are far more complex and not only restricted to the positive effects of fatty acids usage for energy generation. IF interventions involve alternated periods of fasting and feeding, which correspond to different metabolic homeostasis status. In the fasting time, cells adopt a stress-resistance mode through reduction in insulin signaling and overall protein synthesis [3,55,84,87,101,102]. This stress-resistance mode is associated with activation of signaling pathways, which improve mitochondrial function, stress resistance and antioxidant defenses, and also increase autophagy to remove damaged molecules and recycle their components [3,83,103]. In contrast, during the feeding, glucose levels and protein synthesis increase while ketone bodies levels drop, allowing cellular growth and repair [83,84,87,102,103]. Therefore, the maintenance of IF regimens lead to long-term adaptations, most likely through a hormesis effect that improve cellular homeostasis and increase disease resistance [3,84,102,103,104,105,106].

## 5. Conclusions

Overall, we verified that the implementation of the different types of IF protocols has distinct effects. According to data herein presented, ADF and TRF protocols have major beneficial effects in the improvement of dysregulated metabolic conditions. In addition, IF protocols have a major beneficial impact for obese and MetS individuals, through the improvement of adiposity, lipid homeostasis and blood pressure. For T2D individuals, the IF beneficial effects were limited, but associated with their major metabolic dysfunctions. These individuals carefully require consideration of IF protocols, proper medication adjustment and self-monitoring of blood glucose levels to improve results (Graphical abstract).

One of the caveats of this work is related with the high heterogeneity seen, particularly in studies of individuals with obesity and MetS, which can be explained by diversity of IF interventions, as well as, the metabolic status of the individuals, strengthen the notion that IF should be tailored to the individual. Therefore, our data clearly show that IF impacts metabolic homeostasis differently depending on the individual’s basal metabolic status.

In a forward-looking perspective, there is still much research to be carried out in this area, and more studies are needed to fully understand the potential benefits and risks of IF for different populations and under different conditions.

## Figures and Tables

**Table 2:** Characteristics of the participants that integrate the studies included in each intermittent fasting (IF) protocols. Mean (SD) values are shown.

Regime	Weight	BMI	Waist Circumference	Glucose	Total Cholesterol
ADF	94.34 ± 10.39	33.60 ± 2.83	107.43 ± 9.79	105.76 ± 25.79	183.54 ± 14.02
TRF	92.12 ± 7.19	36.09 ± 16.44	105.91 ± 5.09	108.04 ± 24.73	206.98 ± 20.77
RF	83.09 ± 13.15	30.16 ± 11.81	98.29 ± 11.01	156.40 ± 38.90	188.78 ± 24.80

## Data Availability

The data presented in this study are available on request from the corresponding author.

## Supplementary Materials

The following supporting information can be downloaded at: https://www.mdpi.com/article/10.3390/jcm12113699/s1, Table S1: Overview of the main analysis of studies included in qualitative synthesis, Table S2: Assessment of studies quality, Table S3: Analysis of the impact of intermittent fasting on different outcomes and Table S4: Analysis of the impact of intermittent fasting, without the fasting Ramadan studies, on different outcomes.
