# Supplementary material for: Effects of Intermittent Fasting on Regulation of Metabolic Homeostasis: A Systematic Review and Meta-Analysis in Health and Metabolic-Related Disorders
Source: J Clin Med. 2023 May 26;12(11):3699. doi: 10.3390/jcm12113699 (PMC10253889; doi:10.3390/jcm12113699)
Supplement: Supplementary file 1 [file jcm-12-03699-s001.zip › jcm-2229268-supplementary.docx]

**Table S1.** Overview of studies included in qualitative analysis.

| **Study** | **Design** | **Control condition(s)** | **Experimental group condition(s)** | **Protocol** | **Duration-days** | **Age (mean+SD)** | **Number of participants (Gender)** |
| --- | --- | --- | --- | --- | --- | --- | --- |
| Guo Y, et al., 2020 [25] | RCT |  | MetS | 5+2 (NC) | 56 | 42,7 ± 4,1 (C)  40,2 ± 5,7 (EG) | 11(M) 7(W)  10(M) 12(W) |
| Pinto AM, et al., 2019 [8] | RCT | Healthy | Obesity | 5+2 (C) | 28 | 56 ± 8 (C)  50 ± 12 (EG) | 6(M) 16(W)  6(M) 15(W) |
| Carter S, et al., 2019 [6] | RCT | T2D | T2D | 5+2 (NC) | 360 | 61 ± 9,2 (C) | 29(M) 38(W) |
| Parvaresh A, et al, 2019 [33] | RCT | - | MetS | 4+3 (NC) | 56 | 44.6±9.8 | 21(M) 14(W) |
| Gabel K, et al., 2019 [42] | RCT | - | Obesity | 1+1 (NC) | 365 | 43±3 | 9(M) 2(W) |
| Kalam F, et al, 2019 [68] | RCT | - | Overweight | 1+1 (NC) | 365 | 44±1 | 3(M) 18(W) |
| Cho A-Ra, et al, 2019 [40] | RCT | - | Overweight | 4+3 (NC) | 56 | 33.5±5 | 3(M) 6(W) |
| Corley BT, et al., 2018 [9] | RCT | - | T2D | 5+2 (C)  5+2 (NC) | 84 | 62 (44 to 77) **  58 (42 to 74) ** | 11(M) 7(W)  11(M) 8(W) |
| Antony R, et al., 2018 [5] | RCT | Obesity | Obesity | 5+2 (C) | 450 | 48 ± 3 (C) | 6(M) 6(W) |
| Hutchison AT, et al., 2018 [26] | RCT | Overweight or Obesity | Overweight or Obesity | 4+3 (NC) | 70 | 42 ± 4 (EG)  49 ± 2 (C2)  51 ± 2 (EG-IF100)  51 ± 2 (EG-IF70) | 7(M) 8(W)  24(W)  22(W)  22(W) |
| Sundfør TM, et al, 2018 [54] |  | - | Overweight and MetS | 5+2 (NC) | 365 | 49.9±10.1 | 28(M) 26(W) |
| Trepanowski JF, et al, 2018 [55] |  | - | Obesity | 1+1 (NC) | 168 | 46±2 | 3(M) 22(W) |
| Conley M, et al, 2018 [41] | RCT | - | Obesity | 5+2 (NC) | 180 | 68±2.7 | 11(M) |
| Li C, et al., 2017 [29] | RCT | T2D | T2D | 1 week fasting | 7 | 65,4 ± 5,7 (C)  64,7 ± 7 (EG) | 16(NS)  16(NS) |
| Trepanowski JF, et al, 2017 [72] | RCT | - | Obesity | 1+1 (NC) | 180 | 44±10 | 30(M) 4(W) |
| Carter S, et al., 2016 [64] | RCT | Obesity with T2D | Obesity with T2D | 5+2 | 84 | 62 ± 9,1 (C)  61 ± 7,5 (EG) | 16(M) 16(W)  14(M) 17(W) |
| Catenacci VA et al, 2016 [39] | RCT | - | Obesity | 1+1 (NC) | 56 | 39.6±9.5 | 3(M) 10(F) |
| Hoddy KK, et al, 2014 [44] | RCT | - | Obesity | 1+1 (NC) | 70 | ADF-L:45±3  ADF-D:45±3  ADF-SM:46±2 | ADF-L:3(M) 17(W)  ADF-D:4(M) 15(W)  ADF-SM:2(M) 18(W) |
| Furmli S, et al., 2017 [65] | CRS | - | T2D | 4+3 | 250 | 53 | 3(M) |
| Li C, et al, 2013 [69] | CRS | - | Obesity | One week fast | 7 | 49 ± 8.1 | 30(W) |
| Varady KA et al, 2009 [56] | CRS | - | Obesity | 1+1 (NC) | 56 | 46 ± 2.4 | 4(M) 12(W) |
| Kessler CS, et al., 2017 [73] | NRCT | Healthy | Healthy | 6+1 | 56 | 41,36±12,63 (C)  42,45±10,82 (EG) | 7(M) 7(W)  7(M) 15(W) |
| Kotarsky CJ, et al, 2021 [21] | RCT | Overweight or  Obesity | Overweight or  Obesity | 16+8 | 56 | CTL = 44±2  EG = 45±3 | CTL – 9(M) / 1(W)  EG8 – 9(M) / 2(W) |
| de Oliveira Maranhao Pureza IR, et al, 2021 [18] | RCT | Overweight or  Obesity | Overweight or  Obesity | 12+12 | 365 | 61 ± 9 (EG) | 31(M) 39(W) |
| Vidmar AP, et al, 2021 [66] | RCT | Healthy | Obesity | 16+8 | 84 | CTL – 16.38±1.25  TLE + Blinded CGM - 16.16±1.16  TLE + Real-Time CGM  Feedback - 16.8±1.09 | CTL – 3(M)/ 12(W)  TLE + Blinded CGM – 6(M) / 12(W)  TLE + Real-Time CGM Feedback – 5(M)/ 11(W) |
| Lowe DA, et al, 2020 [70] | RCT | - | Obesity | 16+8 | 84 | 46.8±10.8 | 35(M) 24(W) |
| Cienfuegos S, et al., 2020 [31] | RCT | - | Obesity | 20+4  18+6 | 56 | 4h_TRF: 49±2  6h_TRF: 46±3 | 4h_TRF: 2(M) 14(W)  6h_TRF: 1(M) 18(W) |
| Kunduraci YE, et al., 2020 [7] | RCT |  | MetS | 16+8 | 84 | 48,76 ± 2,13 (C) | 15(M) 18(W) |
|  |  |  |  |  |  | 47,44 ± 2,17 (EG) | 16(M) 16(W) |
| Sutton EF, et al., 2018 | RCT | Prediabetes | Prediabetes | 18+6 | 35 | 56 ± 9* | 8(M) |
| Jones R, et al., 2020 [20] | CCS | Healthy | Healthy | 16+8 | 14 | 23 ± 1 (C) | 8(M) |
|  |  |  |  |  |  | 23 ± 1 (EG) | 8(M) |
| Zhao L, et al, 2022 [59] | CRS |  | Overweight | 14+10 | 56 | 63 ± 4 | 15(M) |
| Parr EB, et a., 2020 [22] | CRS | T2D | T2D | 15+9 | 28 | 50,2±8,9 | 9(M) 10(W) |
| Gabel K, et al, 2020 [43] | CRS | - | Obesity | 16+8 | 84 | - | 14(M) |
| Wilkinson MJ, et al, 2020 [57] | CRS | - | MetS | 14+10 | 84 | 59±11.4 | 15(M) 6(W) |
| Anton SD, et al., 2019 [37] | CRS | - | Overweight | 16+8 | 21 | 77.1 | 4(M) 6(W) |
| Kesztyüs D, et al, 2019 [46] | CRS | - | MetS | 16+8 | 90 | 49.1± 12,4 | 9(M) 31(W) |
| Arnason TG, et al., 2017 [60] | CRS | - | T2D | 18-20 hours of fasting per day | 14 | 53,8 ± 9,11 | 1(M) 9(W) |
| Schroder JD, et al., 2019 [23] | NRCT | Obesity | Obesity | 16+8 | 90 | 42,3 ± 3,5 (C)  36,6 ± 1,6 (EG) | 12(W)  20(W) |
| Shafras MS, et al, 2020 [63] | CSS |  | T2D | Ramadan | 29 | 55.54 | 40(M) 64(W) |
| Bener A, et al., 2018 [38] | CSS |  | T2D | Ramadan | 30 | 55.39±15.3 | 593(M) 653(W) |
| Zouhal H, et al, 2020 [34] | RCT | - | Obesity | Ramadan | 30 | 24.5±3.8 | 15(M) |
| Zouhal H, et al, 2020 [35] | RCT | - | Obesity | Ramadan | 30 | 24±3.4 | 14(M) |
| Abdullah K, et al, 2020 [17] | CS | Healthy | Healthy or T2D | Ramadan | 30 | 34,61 ± 4,31 (C)  34,35 ± 3,83 (EG)  50,17 ± 12,95 (EG) | 31(M)  37(M)  30(M) |
| Yeoh ECK, et al, 2015 [58] | CS |  | T2D | Ramadan | 30 | 57±11 | 15(M) 14(W) |
| Feizollahzadeh F et al., 2014 [32] | CS | - | Healthy with overweight | Ramadan | 30 | 47,88 | 70(M) |
| Karatoprak C, et al, 2013 [45] | CS |  | T2D | Ramadan | 30 | 57.4±10.1 | 19(M) 57(W) |
| McNeil J, et al, 2014 [49] | CCS | Healthy | Obesity | Ramadan | 14 | 27 ± 4.5 | 10(M) |
| Hassanein M, et al, 2021 [62] | CRS |  | T2D | Ramadan | 29 | 54 | 180(M) 162(W) |
| Kovil R, et al., 2020 [28] | CRS | - | T2D | Ramadan | 30 | 21-80*** | 25(M) 25(W) |
| Mindikoglu AL, et al., 2020 [74] | CRS | - | Healthy | Ramadan | 30 | 32 | 13(M) 1(W) |
| Harbuwono DS, et al, 2020 [61] | CRS | - | T2D | Ramadan | 29 | 52.5±6.3 | 4(M) 4(W) |
| Faris E, et al., 2019 [19] | CRS | - | Overweight or Obesity | Ramadan | 30 | 36,2 ± 12,5 | 35(M) 22(W) |
| Madkour MI, et al., 2019 [71] | CRS | Overweight or obese | Healthy or Obesity | Ramadan (15+9) | 30 | 29,8 ± 14 (C)  35,72 ± 12,35 (EG) | 6(NS)  34(M) 22(W) |
| Abdessadek M, et al, 2019 [36] | CRS | - | T2D | Ramadan | 30 | - | 57(M) 93(W) |
| Prasetya G, et al., 2018 [30] | CRS | - | Healthy | Ramadan | 29 | 24,3 ± 3,7 | 27(M) |
| Kamble S, et al., 2018 [27] | CRS | - | Healthy | Ramadan | 30 | 20-35*** | 30(NS) |
| Aksungar FB, et al., 2016 [67] | CRS | - | Obesity | Ramadan | 30 | 36 ± 3,12 | 23(W) |
| Sezen Y, et al, 2016 [52] | CRS | - | Obesity | Ramadan | 30 | 37 ± 7 | 70(M) |
| Gnanou JV, et al., 2015 [24] | CRS | - | Healthy | Ramadan | 30 | 19-23*** | 20(M) |
| Sahin SB, et al, 2013 [50] | CRS | - | T2D | Ramadan | 30 | 56.93 ± 9.57 | 40(M) 82(W) |
| Shariatpanahi MV, et al, 2012 [53] | CRS | - | MetS | Ramadan | 30 | 40.14 ± 10.8 | 65(M) |
| Salehi M and Neghab M, 2007 [51] | CRS | - | Obesity | Ramadan | 29 | 23.4 ± 1.3 | 28(M) |
| Khaled MB, et al, 2006 [47] | CRS | - | Obesity | Ramadan | 29 | 23.4 ± 1.3 | 60(W) |
| Khatib FA and Shafagoj YA, 2004 [48] | CRS | - | T2D/Obesity | Ramadan | 29 | 51 ± 10 | 44(M) |

RCT-Randomized controlled trial; CS- Cohort study; CCS-Case control study; CRS- Case report series; NRCT- non-randomized controlled trial; CSS-Cross-sectional study; MS- Metabolic syndrome; CS-central obesity; T2D- diabetes *mellitus* type 2; C-consecutive; NC- Non-consecutive; CTL-Control; EG-Experimental Group; NS-Not specified; TLE - time-limited eating; CGM- continuous glucose monitoring; IF70-Intermittent fasting 70%; IF100-Intermittent fasting 100%; M-Men; W- Women; HOMA - insulin resistance; BMI - Body mass index; *Control is the same group but in a different moment; **mean (range); ***range.

**Table S2**- Assessment of studies’ quality.

| **Randomized controlled trials (RCT) – CASP checklist** | | | | | | | | | | | | | | | | | | | | | | | | | | | | | | | | | | | | | | | | | | | | | | | | | | | | | | |
| --- | --- | --- | --- | --- | --- | --- | --- | --- | --- | --- | --- | --- | --- | --- | --- | --- | --- | --- | --- | --- | --- | --- | --- | --- | --- | --- | --- | --- | --- | --- | --- | --- | --- | --- | --- | --- | --- | --- | --- | --- | --- | --- | --- | --- | --- | --- | --- | --- | --- | --- | --- | --- | --- | --- |
| **Study** | **1** | | | **2** | | | | | **3** | | | | | **4** | | | | | **5** | | | | | **6** | | | **7** | | | | | **8** | | | | | | **9** | | | | | | **10** | | | | | | **11** | | | | **Quality** |
| Kotarsky CJ, et al, 2021 [21] |  | | |  | | | | |  | | | | |  | | | | |  | | | | |  | | |  | | | | |  | | | | | |  | | | | | |  | | | | | |  | | | | Good |
| de Oliveira Maranhao Pureza IR, et al, 2021 [18] |  | | |  | | | | |  | | | | |  | | | | |  | | | | |  | | |  | | | | |  | | | | | |  | | | | | |  | | | | | |  | | | | Good |
| Guo I, et al, 2020 [25] |  | | |  | | | | |  | | | | |  | | | | |  | | | | |  | | |  | | | | |  | | | | | |  | | | | | |  | | | | | |  | | | | Good |
| Kunduraci YE, et al, 2020 [7] |  | | |  | | | | |  | | | | |  | | | | |  | | | | |  | | |  | | | | |  | | | | | |  | | | | | |  | | | | | |  | | | | Good |
| Carter S, et al, 2019 [7] |  | | |  | | | | |  | | | | |  | | | | |  | | | | |  | | |  | | | | |  | | | | | |  | | | | | |  | | | | | |  | | | | Good |
| Corley BT, et al, 2018 [9] |  | | |  | | | | |  | | | | |  | | | | |  | | | | |  | | |  | | | | |  | | | | | |  | | | | | |  | | | | | |  | | | | Good |
| Hutchison AT, et al, 2018 [26] |  | | |  | | | | |  | | | | |  | | | | |  | | | | |  | | |  | | | | |  | | | | | |  | | | | | |  | | | | | |  | | | | Good |
| Li C, et al, 2017 [29] |  | | |  | | | | |  | | | | |  | | | | |  | | | | |  | | |  | | | | |  | | | | | |  | | | | | |  | | | | | |  | | | | Moderate |
| Pinto AM, et al, 2019 [8] |  | | |  | | | | |  | | | | |  | | | | |  | | | | |  | | |  | | | | |  | | | | | |  | | | | | |  | | | | | |  | | | | Good |
| Sutton EF, et al, 2018 |  | | |  | | | | |  | | | | |  | | | | |  | | | | |  | | |  | | | | |  | | | | | |  | | | | | |  | | | | | |  | | | | Good |
| Antony R, et al, 2018 [5] |  | | |  | | | | |  | | | | |  | | | | |  | | | | |  | | |  | | | | |  | | | | | |  | | | | | |  | | | | | |  | | | | Good |
| Carter S, et al, 2016 [6] |  | | |  | | | | |  | | | | |  | | | | |  | | | | |  | | |  | | | | |  | | | | | |  | | | | | |  | | | | | |  | | | | Good |
| Vidmar AP, et al, 2021 [66] |  | | |  | | | | |  | | | | |  | | | | |  | | | | |  | | |  | | | | |  | | | | | |  | | | | | |  | | | | | |  | | | | Good |
| Lowe DA, et al., 2020 [70] |  | | |  | | | | |  | | | | |  | | | | |  | | | | |  | | |  | | | | |  | | | | | |  | | | | | |  | | | | | |  | | | | Good |
| Zouhal H, et al., 2020 [34] |  | | |  | | | | |  | | | | |  | | | | |  | | | | |  | | |  | | | | |  | | | | | |  | | | | | |  | | | | | |  | | | | Good |
| Zouhal H, et al., 2020 (2) [35] |  | | |  | | | | |  | | | | |  | | | | |  | | | | |  | | |  | | | | |  | | | | | |  | | | | | |  | | | | | |  | | | | Good |
| Cienfuegos S, et al., 2020 [31] |  | | |  | | | | |  | | | | |  | | | | |  | | | | |  | | |  | | | | |  | | | | | |  | | | | | |  | | | | | |  | | | | Good |
| Parvaresh A, et al., 2019 [33] |  | | |  | | | | |  | | | | |  | | | | |  | | | | |  | | |  | | | | |  | | | | | |  | | | | | |  | | | | | |  | | | | Good |
| Gabel K, et al., 2019 [42] |  | | |  | | | | |  | | | | |  | | | | |  | | | | |  | | |  | | | | |  | | | | | |  | | | | | |  | | | | | |  | | | | Good |
| Kalam F, et al., 2019 [68] |  | | |  | | | | |  | | | | |  | | | | |  | | | | |  | | |  | | | | |  | | | | | |  | | | | | |  | | | | | |  | | | | Good |
| Cho A-Ra, et al., 2019 [40] |  | | |  | | | | |  | | | | |  | | | | |  | | | | |  | | |  | | | | |  | | | | | |  | | | | | |  | | | | | |  | | | | Good |
| Sundfør TM, et al., 2018 [54] |  | | |  | | | | |  | | | | |  | | | | |  | | | | |  | | |  | | | | |  | | | | | |  | | | | | |  | | | | | |  | | | | Good |
| Trepanowski JF, et al., 2018 [55] |  | | |  | | | | |  | | | | |  | | | | |  | | | | |  | | |  | | | | |  | | | | | |  | | | | | |  | | | | | |  | | | | Good |
| Conley M, et al., 2018 [41] |  | | |  | | | | |  | | | | |  | | | | |  | | | | |  | | |  | | | | |  | | | | | |  | | | | | |  | | | | | |  | | | | Good |
| Trepanowski JF, et al., 2017 [72] |  | | |  | | | | |  | | | | |  | | | | |  | | | | |  | | |  | | | | |  | | | | | |  | | | | | |  | | | | | |  | | | | Good |
| Catenacci VA, et al., 2016 [39] |  | | |  | | | | |  | | | | |  | | | | |  | | | | |  | | |  | | | | |  | | | | | |  | | | | | |  | | | | | |  | | | | Good |
| Hoddy KK, et al, 2014 [44] |  | | |  | | | | |  | | | | |  | | | | |  | | | | |  | | |  | | | | |  | | | | | |  | | | | | |  | | | | | |  | | | | Good |
| **Cohort studies (CS) - CASP checklist** | | | | | | | | | | | | | | | | | | | | | | | | | | | | | | | | | | | | | | | | | | | | | | | | | | | | | | |
|  | **1** | **2** | | | | | **3** | | | | **4** | | | | **5a** | | | **5b** | | | | | **6a** | | **6b** | | | **7** | | | | | **8** | | | | **9** | | | | | **10** | | | | | **11** | | | | | | **12** |  |
| Abdullah K, et al, 2020 [17] |  |  | | | | |  | | | |  | | | |  | | |  | | | | |  | |  | | |  | | | | |  | | | |  | | | | |  | | | | |  | | | | | |  | Moderate |
| Feizollahzadeh S, et al, 2014 [32] |  |  | | | | |  | | | |  | | | |  | | |  | | | | |  | |  | | |  | | | | |  | | | |  | | | | |  | | | | |  | | | | | |  | Good |
| Yeoh, Ester C K et al., 2015 [58] |  |  | | | | |  | | | |  | | | |  | | |  | | | | |  | |  | | |  | | | | |  | | | |  | | | | |  | | | | |  | | | | | |  | Moderate |
| Karatoprak C, et al, 2013 [45] |  |  | | | | |  | | | |  | | | |  | | |  | | | | |  | |  | | |  | | | | |  | | | |  | | | | |  | | | | |  | | | | | |  | Good |
| **Case-control studies (CCS) - CASP checklist** | | | | | | | | | | | | | | | | | | | | | | | | | | | | | | | | | | | | | | | | | | | | | | | | | | | | | | |
|  | **1** | | **2** | | | | | **3** | | | | **4** | | | | | **5** | | | | | **6a** | | | **6b** | | | | **7** | | | | | **8** | | | | | **9** | | | | | | **10** | | | | | | **11** | | |  |
| Jones R, et al, 2020 [20] |  | |  | | | | |  | | | |  | | | | |  | | | | |  | | |  | | | |  | | | | |  | | | | |  | | | | | |  | | | | | |  | | | Good |
| McNeil J, et al, 2014 [49] |  | |  | | | | |  | | | |  | | | | |  | | | | |  | | |  | | | |  | | | | |  | | | | |  | | | | | |  | | | | | |  | | | Moderate |
| **Case report series (CRS) – Joanna Briggs** | | | | | | | | | | | | | | | | | | | | | | | | | | | | | | | | | | | | | | | | | | | | | | | | | | | | | | |
|  | **1** | | | | **2** | | | | | **3** | | | | | | **4** | | | | | **5** | | | | **6** | | | | | **7** | | | | | | **8** | | | | | | | **9** | | | | | | **10** | | | | |  |
| Faris E, et al, 2019 [19] |  | | | |  | | | | |  | | | | | |  | | | | |  | | | |  | | | | |  | | | | | |  | | | | | | |  | | | | | |  | | | | | Moderate |
| Parr EB, et al, 2020 [22] |  | | | |  | | | | |  | | | | | |  | | | | |  | | | |  | | | | |  | | | | | |  | | | | | | |  | | | | | |  | | | | | Good |
| Kovil R, et al, 2020 [28] |  | | | |  | | | | |  | | | | | |  | | | | |  | | | |  | | | | |  | | | | | |  | | | | | | |  | | | | | |  | | | | | Good |
| Prasetya G, et al, 2018 [30] |  | | | |  | | | | |  | | | | | |  | | | | |  | | | |  | | | | |  | | | | | |  | | | | | | |  | | | | | |  | | | | | Good |
| Kamble S, et al, 2018 [27] |  | | | |  | | | | |  | | | | | |  | | | | |  | | | |  | | | | |  | | | | | |  | | | | | | |  | | | | | |  | | | | | Good |
| Arnason FB, et al, 2017 [60] |  | | | |  | | | | |  | | | | | |  | | | | |  | | | |  | | | | |  | | | | | |  | | | | | | |  | | | | | |  | | | | | Good |
| Gnanou JV, et al, 2015 [24] |  | | | |  | | | | |  | | | | | |  | | | | |  | | | |  | | | | |  | | | | | |  | | | | | | |  | | | | | |  | | | | | Good |
| Mindikoglu AL, et al, 2020 [74] |  | | | |  | | | | |  | | | | | |  | | | | |  | | | |  | | | | |  | | | | | |  | | | | | | |  | | | | | |  | | | | | Moderate |
| Madkour MI, et al, 2019 [71] |  | | | |  | | | | |  | | | | | |  | | | | |  | | | |  | | | | |  | | | | | |  | | | | | | |  | | | | | |  | | | | | Good |
| Aksungar FB, et al, 2016 [67] |  | | | |  | | | | |  | | | | | |  | | | | |  | | | |  | | | | |  | | | | | |  | | | | | | |  | | | | | |  | | | | | Good |
| Furmli S, et al, 2017 [65] |  | | | |  | | | | |  | | | | | |  | | | | |  | | | |  | | | | |  | | | | | |  | | | | | | |  | | | | | |  | | | | | Weak |
| Zhao L, et al., 2022 [59] |  | | | |  | | | | |  | | | | | |  | | | | |  | | | |  | | | | |  | | | | | |  | | | | | | |  | | | | | |  | | | | | Moderate |
| Hassanein M, et al., 2021 [62] |  | | | |  | | | | |  | | | | | |  | | | | |  | | | |  | | | | |  | | | | | |  | | | | | | |  | | | | | |  | | | | | Good |
| Harbuwono DS, et al., 2020 [61] |  | | | |  | | | | |  | | | | | |  | | | | |  | | | |  | | | | |  | | | | | |  | | | | | | |  | | | | | |  | | | | | Moderate |
| Gabel K, et al., 2020 [43] |  | | | |  | | | | |  | | | | | |  | | | | |  | | | |  | | | | |  | | | | | |  | | | | | | |  | | | | | |  | | | | | Moderate |
| Wilkinson MJ, et al., 2020 [57] |  | | | |  | | | | |  | | | | | |  | | | | |  | | | |  | | | | |  | | | | | |  | | | | | | |  | | | | | |  | | | | | Good |
| Kesztyüs D, et al., 2019 [46] |  | | | |  | | | | |  | | | | | |  | | | | |  | | | |  | | | | |  | | | | | |  | | | | | | |  | | | | | |  | | | | | Moderate |
| Anton SD, et al., 2019 [37] |  | | | |  | | | | |  | | | | | |  | | | | |  | | | |  | | | | |  | | | | | |  | | | | | | |  | | | | | |  | | | | | Moderate |
| Abdessadek M, et al., 2019 [36] |  | | | |  | | | | |  | | | | | |  | | | | |  | | | |  | | | | |  | | | | | |  | | | | | | |  | | | | | |  | | | | | Good |
| Sezen Y, et al., 2016 [52] |  | | | |  | | | | |  | | | | | |  | | | | |  | | | |  | | | | |  | | | | | |  | | | | | | |  | | | | | |  | | | | | Good |
| Sahin SB, et al, 2013 [50] |  | | | |  | | | | |  | | | | | |  | | | | |  | | | |  | | | | |  | | | | | |  | | | | | | |  | | | | | |  | | | | | Moderate |
| Shariatpanahi MV, et al, 2012 [53] |  | | | |  | | | | |  | | | | | |  | | | | |  | | | |  | | | | |  | | | | | |  | | | | | | |  | | | | | |  | | | | | Moderate |
| Salehi M and Neghab M, 2007 [51] |  | | | |  | | | | |  | | | | | |  | | | | |  | | | |  | | | | |  | | | | | |  | | | | | | |  | | | | | |  | | | | | Weak |
| Varady KA, et al, 2009 [56] |  | | | |  | | | | |  | | | | | |  | | | | |  | | | |  | | | | |  | | | | | |  | | | | | | |  | | | | | |  | | | | | Good |
| Khatib FA and Shafagoj YA, 2004 [48] |  | | | |  | | | | |  | | | | | |  | | | | |  | | | |  | | | | |  | | | | | |  | | | | | | |  | | | | | |  | | | | | Moderate |
| Khaled MB, et al, 2006 [47] |  | | | |  | | | | |  | | | | | |  | | | | |  | | | |  | | | | |  | | | | | |  | | | | | | |  | | | | | |  | | | | | Moderate |
| Li C, et al, 2013 [69] |  | | | |  | | | | |  | | | | | |  | | | | |  | | | |  | | | | |  | | | | | |  | | | | | | |  | | | | | |  | | | | | Moderate |
| **Non-randomized controlled trials (NRCT)– MINORS** | | | | | | | | | | | | | | | | | | | | | | | | | | | | | | | | | | | | | | | | | | | | | | | | | | | | | | |
|  | **1** | | **2** | | | | | **3** | | | | **4** | | | | | **5** | | | | | **6** | | | **7** | | | | **8** | | | | | | **9** | | | | | **10** | | | | | | **11** | | | | | | **12** | |  |
| Schroder JD, et al, 2021 [23] |  | |  | | | | |  | | | |  | | | | |  | | | | |  | | |  | | | |  | | | | | |  | | | | |  | | | | | |  | | | | | |  | | Good |
| Kessler CS, et al, 2017 [73] |  | |  | | | | |  | | | |  | | | | |  | | | | |  | | |  | | | |  | | | | | |  | | | | |  | | | | | |  | | | | | |  | | Good |
| **Cross sectional studies (CSS) – Joanna Briggs** | | | | | | | | | | | | | | | | | | | | | | | | | | | | | | | | | | | | | | | | | | | | | | | | | | | | | | |
|  | **1** | | | | | **2** | | | | | | | **3** | | | | | | | **4** | | | | | | **5** | | | | | **6** | | | | | | | | | | **7** | | | | | | | **8** | | | | | |  |
| Shafras MS, et al., 2020 [63] |  | | | | |  | | | | | | |  | | | | | | |  | | | | | |  | | | | |  | | | | | | | | | |  | | | | | | |  | | | | | | Good |
| Bener A, et al, 2018 [38] |  | | | | |  | | | | | | |  | | | | | | |  | | | | | |  | | | | |  | | | | | | | | | |  | | | | | | |  | | | | | | Moderate |

**Table S3**. Analysis of the impact of intermittent fasting on different outcomes in healthy individuals and/or individuals with metabolic related disorders as type 2 diabetes mellitus (T2D), metabolic syndrome (MetS) or obesity.

|  | **Moderators** | **K^1^** | **Point estimate** | **CI lower** | **CI upper** | ***P*-value** | **Heterogeneity** | | |
| --- | --- | --- | --- | --- | --- | --- | --- | --- | --- |
|  |  |  |  |  |  |  | Q-value | *P*-value | I-squared |
| **Adiposity** | **Weight (Kg)** |  |  |  |  |  |  |  |  |
|  | All studies | 39 | 3.54 | 2.56 | 4.52 | <0.00001 | 106.95 | <0.00001 | 62.00 |
|  | Healthy | 6 | 2.09 | 0.62 | 3.56 | 0.005 | 4.50 | 0.48 | 0.00 |
|  | Obesity | 21 | 4.14 | 3.02 | 5.26 | <0.00001 | 47.56 | 0.0008 | 56.00 |
|  | T2D | 8 | 1.63 | -0.37 | 3.64 | 0.11 | 2.07 | 0.98 | 0.00 |
|  | MetS | 5 | 6.13 | 3.29 | 8.97 | <0.00001 | 5.77 | 0.22 | 31.00 |
|  | **BMI (Kg/m^2^)** |  |  |  |  |  |  |  |  |
|  | All studies | 38 | 1.34 | 1.00 | 1.69 | <0.00001 | 129.71 | <0.00001 | 68.00 |
|  | Healthy | 5 | 0.70 | 0.32 | 1.09 | 0.0004 | 2.46 | 0.65 | 0.00 |
|  | Obesity | 17 | 1.69 | 1.13 | 2.26 | <0.00001 | 50.77 | <0.00001 | 68.00 |
|  | T2D | 12 | 1.26 | 0.99 | 1.53 | <0.00001 | 7.68 | 0.91 | 0.00 |
|  | MetS | 5 | 1.67 | 0.38 | 2.96 | 0.01 | 15.12 | 0.004 | 74.00 |
|  | **Waist circumference (cm)** |  |  |  |  |  |  |  |  |
|  | All studies | 21 | 3.85 | 2.60 | 5.10 | <0.00001 | 65.84 | <0.00001 | 65.00 |
|  | Healthy | 3 | 1.32 | -1.34 | 3.98 | 0.33 | 1.04 | 0.59 | 0.00 |
|  | Obesity | 10 | 5.09 | 3.40 | 6.79 | <0.00001 | 41.82 | <0.00001 | 76.00 |
|  | T2D | 5 | 1.73 | -0.95 | 4.42 | 0.21 | 1.20 | 0.88 | 0.00 |
|  | MetS | 5 | 3.22 | 1.33 | 5.11 | 0.0008 | 0.95 | 0.92 | 0.00 |
| **Lipid homeostasis** | **HDL-c (mg/dL)** |  |  |  |  |  |  |  |  |
|  | All studies | 33 | 1.18 | 0.71 | 1.64 | <0.00001 | 1098.34 | <0.00001 | 96.00 |
|  | Healthy | 5 | -0.91 | -2.90 | 1. 08 | 0.37 | 5.52 | 0.24 | 27.00 |
|  | Obesity | 15 | 1.50 | 0.84 | 2.16 | <0.00001 | 768.34 | <0.00001 | 98.00 |
|  | T2D | 10 | 1.74 | 0.05 | 3.43 | 0.04 | 97.06 | <0.00001 | 89.00 |
|  | MetS | 5 | -0.75 | -3.78 | 2.28 | 0.63 | 22.82 | 0.0001 | 82.00 |
|  | **LDL-c (mg/dL)** |  |  |  |  |  |  |  |  |
|  | All studies | 30 | 2.23 | -0.82 | 5.28 | 0.15 | 45249.14 | <0.00001 | 100.00 |
|  | Healthy | 5 | -4.84 | -11.31 | 1.53 | 0.14 | 1.54 | 0.82 | 0.00 |
|  | Obesity | 13 | 4.12 | 0.83 | 7.40 | 0.21 | 27.03 | <0.00001 | 85.00 |
|  | T2D | 9 | -5.11 | -8.28 | -1.95 | 0.002 | 21.66 | 0.02 | 54.00 |
|  | MetS | 5 | 10.26 | -5.96 | 26.49 | 0.21 | 27.03 | <0.00001 | 85.00 |
|  | **Total cholesterol (mg/dL)** |  |  |  |  |  |  |  |  |
|  | All studies | 32 | 14.30 | 11.10 | 17.51 | <0.00001 | 44419.38 | <0.00001 | 100.00 |
|  | Healthy | 5 | 15.85 | -56.12 | 87.81 | 0.67 | 442.03 | <0.00001 | 99.00 |
|  | Obesity | 15 | 12.53 | 9.04 | 16.03 | <0.00001 | 22309.98 | <0.00001 | 100.00 |
|  | T2D | 9 | 12.83 | -9.74 | 35.40 | 0.27 | 1436.28 | <0.00001 | 99.00 |
|  | MetS | 5 | 17.42 | 5.18 | 29.67 | 0.005 | 12.92 | 0.01 | 69.00 |
|  | **Triglycerides (mg/dL)** |  |  |  |  |  |  |  |  |
|  | All studies | 35 | 13.29 | 2.53 | 24.05 | 0.02 | 2029.14 | <0.00001 | 98.00 |
|  | Healthy | 6 | -0.86 | -9.83 | 8.12 | 0.85 | 13.60 | 0.02 | 63.00 |
|  | Obesity | 15 | 19.05 | -0.09 | 38.20 | 0.05 | 1683.28 | <0.00001 | 99.00 |
|  | T2D | 11 | 6.28 | 1.29 | 11.26 | 0.01 | 14.48 | 0.27 | 17.00 |
|  | MetS | 5 | 20.70 | -4.81 | 46.20 | 0.11 | 14.13 | 0.007 | 72.00 |
| **Insulin homeostasis** | **Fasting glucose (mg/dL)** |  |  |  |  |  |  |  |  |
|  | All studies | 31 | 8.86 | 5.92 | 11.81 | <0.00001 | 2914.14 | <0.00001 | 99.00 |
|  | Healthy | 4 | -1.36 | -3.44 | 0.72 | 0.20 | 9.62 | 0.02 | 69.00 |
|  | Obesity | 14 | 2.81 | 0.52 | 5.10 | 0.02 | 20.32 | 0.0001 | 94.00 |
|  | T2D | 10 | 22.46 | 14.33 | 30.58 | <0.00001 | 569.35 | <0.00001 | 98.00 |
|  | MetS | 4 | 12.28 | 5.38 | 19.17 | 0.0005 | 20.32 | 0.0001 | 85.00 |
|  | **Fasting insulin (mU/L)** |  |  |  |  |  |  |  |  |
|  | All studies | 24 | 1.62 | 0.17 | 3.08 | 0.03 | 524.38 | <0.00001 | 95.00 |
|  | Healthy | 5 | 0.14 | -0.95 | 1. 22 | 0.80 | 3.72 | 0.44 | 0.00 |
|  | Obesity | 12 | 1.31 | -0.57 | 3.18 | 0.17 | 393.87 | <0.00001 | 97.00 |
|  | T2D | 5 | 2.06 | 0.40 | 3.71 | 0.02 | 2.55 | 0.64 | 0.00 |
|  | MetS | 4 | 4.87 | 0.72 | 9.01 | 0.02 | 15.88 | 0.001 | 81.00 |
|  | **HOMA-IR** |  |  |  |  |  |  |  |  |
|  | All studies | 16 | 0.69 | 0.10 | 1.27 | 0.02 | 781.21 | <0.00001 | 98.00 |
|  | Healthy | 4 | -0.05 | -0.26 | 0.16 | 0.63 | 3.61 | 0.31 | 17.00 |
|  | Obesity | 8 | 0.99 | 0.07 | 1.91 | 0.03 | 663.41 | <0.00001 | 99.00 |
|  | T2D | 3 | -0.31 | -1.29 | 0.66 | 0.53 | 2.27 | 0.32 | 12.00 |
|  | MetS | 3 | 1.21 | 0.93 | 1.49 | <0.00001 | 1.92 | 0.38 | 0.00 |
| **Blood pressure** | **SBP (mmHg)** |  |  |  |  |  |  |  |  |
|  | All studies | 25 | 3.87 | 2.29 | 5.46 | <0.00001 | 219.55 | <0.00001 | 86.00 |
|  | Healthy | 1 | -0.22 | -1.92 | 1.48 | 0.80 | - | - | - |
|  | Obesity | 13 | 3.87 | 2.19 | 5.56 | <0.00001 | 71.95 | <0.00001 | 79.00 |
|  | T2D | 8 | 3.25 | -0.62 | 7.22 | 0.11 | 77.23 | <0.00001 | 88.00 |
|  | MetS | 4 | 7.47 | 5.78 | 9.16 | <0.00001 | 3.11 | 0.38 | 4.00 |
|  | **DBP (mmHg)** |  |  |  |  |  |  |  |  |
|  | All studies | 25 | 2.33 | 1.52 | 3.13 | <0.00001 | 161.89 | <0.00001 | 81.00 |
|  | Healthy | 1 | -0.67 | -2.42 | 1.08 | 0.45 | - | - | - |
|  | Obesity | 13 | 2.60 | 1.93 | 3.28 | <0.00001 | 32.13 | 0.006 | 53.00 |
|  | T2D | 8 | 1.29 | 0.01 | 2.57 | 0.05 | 22.67 | 0.007 | 60.00 |
|  | MetS | 4 | 4.97 | 3.84 | 6.10 | <0.00001 | 3.23 | 0.36 | 7.00 |

^1^k- number of studies; MetS- Metabolic syndrome; T2D- diabetes *mellitus* type 2; HOMA - insulin resistance; BMI - Body mass index; SBP- Systolic Blood Pressure; DBP- Diastolic Blood Pressure.

**Table S4.** Analysis of the impact of intermittent fasting, without the fasting Ramadan studies, on different outcomes in healthy individuals and/or individuals with metabolic related disorders as type 2 diabetes mellitus (T2D), metabolic syndrome (MetS) or obesity.

|  | **Moderators** | **K^1^** | **Point estimate** | **CI lower** | **CI upper** | ***P*-value** | **Heterogeneity** | | |
| --- | --- | --- | --- | --- | --- | --- | --- | --- | --- |
|  |  |  |  |  |  |  | Q-value | *P*-value | I-squared |
| **Adiposity** | **Weight (Kg)** |  |  |  |  |  |  |  |  |
|  | All studies | 25 | 4.27 | 3.04 | 5.51 | <0.00001 | 84.84 | <0.00001 | 69.00 |
|  | Healthy | 1 | 1.04 | -1.89 | 3.97 | 0.49 | - | - | - |
|  | Obesity | 16 | 4.26 | 3.00 | 5.55 | <0.00001 | 45.62 | 0.0002 | 63.00 |
|  | T2D | 4 | 3.74 | 0.00 | 7.48 | 0.05 | 0.06 | 1 | 0.00 |
|  | MetS | 4 | 6.03 | 2.80 | 9.26 | 0.0003 | 5.24 | 0.15 | 43.00 |
|  | **BMI (Kg/m^2^)** |  |  |  |  |  |  |  |  |
|  | All studies | 12 | 1.48 | 0.59 | 2.36 | 0.001 | 46.05 | <0.00001 | 74.00 |
|  | Healthy | 0 | - | - | - | - | - | - | - |
|  | Obesity | 4 | 1.04 | 0.56 | 1.51 | 0.001 | 2.92 | 0.40 | 0.00 |
|  | T2D | 5 | 1.13 | 0.15 | 2.11 | 0.002 | 0.99 | 0.96 | 0.00 |
|  | MetS | 3 | 2.17 | 0.79 | 3.56 | 0.002 | 5.88 | 0.05 | 66.00 |
|  | **Waist circumference (cm)** |  |  |  |  |  |  |  |  |
|  | All studies | 14 | 5.15 | 3.72 | 6.58 | <0.00001 | 39.98 | 0.0003 | 65.00 |
|  | Healthy | 0 | - | - | - | - | - | - | - |
|  | Obesity | 8 | 5.54 | 3.78 | 7.31 | <0.00001 | 37.50 | <0.00001 | 79.00 |
|  | T2D | 3 | 3.60 | -0.99 | 8.19 | 0.12 | 0.21 | 0.9 | 0.00 |
|  | MetS | 3 | 3.98 | 0.99 | 7.06 | 0.01 | 0.39 | 0.82 | 0.00 |
| **-Lipid homeostasis** | **HDL-c (mg/dL)** |  |  |  |  |  |  |  |  |
|  | All studies | 21 | 1.61 | 1.09 | 2.13 | <0.00001 | 894.24 | <0.00001 | 97.00 |
|  | Healthy | 0 | - | - | - | - | - | - | - |
|  | Obesity | 13 | 1.47 | 0.80 | 2.15 | <0.00001 | 762.58 | <0.00001 | 98.00 |
|  | T2D | 6 | 1.16 | -1.37 | 3.68 | 0.37 | 11.95 | 0.06 | 50.00 |
|  | MetS | 3 | -0.3 | -1.39 | 0.79 | 0.58 | 2.09 | 0.35 | 4.00 |
|  | **LDL-c (mg/dL)** |  |  |  |  |  |  |  |  |
|  | All studies | 9 | 3.37 | -10.38 | 17.12 | 0.63 | 408.99 | <0.00001 | 98.00 |
|  | Healthy | 0 | - | - | - | - | - | - | - |
|  | Obesity | 1 | -8.80 | -14.40 | -3.20 | 0.01 | 22335.31 | <0.00001 | 100.00 |
|  | T2D | 4 | -3.03 | -10.89 | 4.84 | 0.45 | 5.54 | 0.24 | 28.00 |
|  | MetS | 4 | 9.64 | -9.35 | 28.63 | 0.32 | 25.59 | <0.00001 | 88.00 |
|  | **Total cholesterol (mg/dL)** |  |  |  |  |  |  |  |  |
|  | All studies | 19 | 9.62 | 5.63 | 13.61 | <0.00001 | 43122.47 | <0.00001 | 100.00 |
|  | Healthy | 0 | - | - | - | - | - | - | - |
|  | Obesity | 12 | 9.41 | 5.73 | 13.08 | <0.00001 | 22166.75 | <0.00001 | 100.00 |
|  | T2D | 4 | -0.36 | -7.09 | 6.38 | 0.92 | 2.39 | 0.3 | 16.00 |
|  | MetS | 3 | 17.93 | 4.03 | 31.84 | 0.001 | 11.58 | 0.009 | 74.00 |
|  | **Triglycerides (mg/dL)** |  |  |  |  |  |  |  |  |
|  | All studies | 21 | 18.27 | 3.15 | 33.39 | 0.02 | 1916.17 | <0.00001 | 99.00 |
|  | Healthy | 1 | 1.04 | -1.89 | 3.97 | 0.49 | - | - | - |
|  | Obesity | 12 | 19.38 | -1.52 | 40.29 | 0.07 | 1665.39 | <0.00001 | 99.00 |
|  | T2D | 3 | 4.44 | -7.70 | 16.59 | 0.47 | 1.10 | 0.95 | 0.00 |
|  | MetS | 5 | 32.55 | 5.70 | 59.39 | 0.002 | 5.42 | 0.07 | 63.00 |
| **Insulin homeostasis** | **Fasting glucose (mg/dL)** |  |  |  |  |  |  |  |  |
|  | All studies | 18 | 5.28 | 1.77 | 8.79 | 0.003 | 1156.70 | <0.00001 | 98.00 |
|  | Healthy | 1 | 0.54 | -1.33 | 2.41 | 0.57 | - | - | - |
|  | Obesity | 11 | 2.90 | 0.45 | 5.34 | 0.02 | 225.46 | <0.00001 | 95.00 |
|  | T2D | 2 | 11.65 | 3.18 | 20.11 | 0.007 | 137.18 | <0.00001 | 98.00 |
|  | MetS | 4 | 10.37 | 0.11 | 20.62 | 0.05 | 16.05 | <0.00001 | 94.00 |
|  | **Fasting insulin (mU/L)** |  |  |  |  |  |  |  |  |
|  | All studies | 17 | 2.44 | 0.63 | 4.26 | 0.008 | 467.83 | <0.00001 | 96.00 |
|  | Healthy | 1 | 1.58 | -8.05 | 0.21 | 0.75 | - | - | - |
|  | Obesity | 10 | 1.79 | -0.15 | 3.72 | 0.07 | 353.82 | <0.00001 | 97.00 |
|  | T2D | 3 | 3.86 | -0.14 | 7.86 | 0.06 | 0 | 0.97 | 0.00 |
|  | MetS | 3 | 5.10 | 0.27 | 9.93 | 0.04 | 14.93 | 0.0006 | 87.00 |
|  | **HOMA** |  |  |  |  |  |  |  |  |
|  | All studies | 10 | 0.79 | 0.15 | 1.43 | 0.01 | 354.84 | <0.00001 | 97.00 |
|  | Healthy | 0 | - | - | - | - | - | - | - |
|  | Obesity | 6 | 0.72 | -0.06 | 1.51 | 0.07 | 334.0 | <0.00001 | 98.00 |
|  | T2D | 2 | 0.80 | -1.09 | 2.70 | 0.41 | 0.3 | 0.58 | 0.00 |
|  | MetS | 2 | 1.12 | 0.61 | 1.62 | <0.00001 | 1.86 | 0.17 | 46.00 |
| **Blood pressure** | **SBP (mmHg)** |  |  |  |  |  |  |  |  |
|  | All studies | 19 | 5.00 | 3.48 | 6.52 | <0.00001 | 83.25 | <0.00001 | 74.00 |
|  | Healthy | 0 | - | - | - | - | - | - | - |
|  | Obesity | 11 | 4.31 | 2.54 | 6.08 | <0.00001 | 66.82 | <0.00001 | 79.00 |
|  | T2D | 5 | 5.82 | 1.73 | 9.92 | 0.005 | 2.98 | 0.70 | 0.00 |
|  | MetS | 3 | 8.22 | 4.64 | 11.79 | <0.00001 | 2.70 | 0.26 | 26.00 |
|  | **DBP (mmHg)** |  |  |  |  |  |  |  |  |
|  | All studies | 19 | 3.08 | 2.37 | 3.79 | <0.00001 | 60.08 | <0.00001 | 63.00 |
|  | Healthy | 0 | - | - | - | - | - | - | - |
|  | Obesity | 11 | 2.75 | 2.09 | 3.42 | <0.00001 | 27.61 | 0.01 | 53.00 |
|  | T2D | 5 | 3.19 | 0.06 | 6.32 | 0.05 | 7.80 | 0.17 | 36.00 |
|  | MetS | 3 | 5.17 | 3.06 | 7.28 | <0.00001 | 2.96 | 0.23 | 32.00 |

^1^k- number of studies; MetS- Metabolic syndrome; T2D- diabetes *mellitus* type 2; HOMA - insulin resistance; BMI - Body mass Index; SBP- Systolic Blood Pressure; DBP- Diastolic Blood Pressure.
